# Unravelling CO Activation on Flat and Stepped Co Surfaces: A Molecular Orbital Analysis

**DOI:** 10.1021/acs.jpcc.4c00144

**Published:** 2024-05-23

**Authors:** Rozemarijn D.E. Krösschell, Emiel J.M. Hensen, Ivo A.W. Filot

**Affiliations:** Laboratory of Inorganic Materials & Catalysis, Department of Chemical Engineering and Chemistry, Eindhoven University of Technology, PO Box 513, Eindhoven 5600 MB, The Netherlands

## Abstract

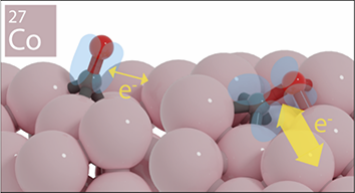

Structure sensitivity in heterogeneous catalysis dictates the overall activity and selectivity of a catalyst whose origins lie in the atomic configurations of the active sites. We explored the influence of the active site geometry on the dissociation activity of CO by investigating the electronic structure of CO adsorbed on 12 different Co sites and correlating its electronic structure features to the corresponding C–O dissociation barrier. By including the electronic structure analyses of CO adsorbed on step-edge sites, we expand upon the current models that primarily pertain to flat sites. The most important descriptors for activation of the C–O bond are the decrease in electron density in CO’s 1π orbital , the occupation of 2π anti-bonding orbitals and the redistribution of electrons in the 3σ orbital. The enhanced weakening of the C–O bond that occurs when CO adsorbs on sites with a step-edge motif as compared to flat sites is caused by a distancing of the 1π orbital with respect to Co. This distancing reduces the electron–electron repulsion with the Co *d*-band. These results deepen our understanding of the electronic phenomena that enable the breaking of a molecular bond on a metal surface.

## 1 Introduction

Structure sensitivity is a phenomenon encountered in heterogeneous catalysis, where the reaction rate strongly depends on the size of the nanoparticle. The contemporary view is that the size of the nanoparticles determines the abundance and stability of the sites required for the activation of a critical bond in a reactant or intermediate. This concept was pioneered a little over half a century ago by van Boudart.^1^ Not only the abundance and geometry of the active sites itself determine the reactivity of the nanoparticle with adsorbates but also the coordination number of the metal atoms. No̷rskov et al.^2^ showed that a decreasing coordination number leads to a decrease of the *d*-bandwidth and an increase of the *d*-band center. This can result in strong molecular chemisorption on metal atoms with a low coordination number, e.g., on very small nanoparticles, at the metal–support interface, or on step sites. Several important reactions in heterogeneous catalysis show a strong structure sensitivity relationship. For example, for steam methane reforming it is found that decreasing the nanoparticle size improves the activity of this reaction as smaller particles expose more kink and corner sites which are instrumental in the activation of the C–H σ-bond.^3−5^ In contrast, for ammonia synthesis,^6−8^ Fischer–Tropsch synthesis^9−14^ and CO_2_ methanation,^15−18^ it is found that the turnover frequency (TOF) increases with increasing nanoparticle size. For these reactions, the activation of a π-bond is critical, which requires the availability of step-edge or B_5_ sites (as defined by Van Hardeveld et al.^19^), whose abundance increases with increasing particle size.

The structure sensitivity relationship as found in heterogeneous catalysis already points out the fact that the specific topology of an active site plays a crucial role in the activation of chemical bonds. Consequently, vast differences in activation energies are observed as a function of the active site configuration.^20^ In this study, we aim to understand the underlying electronic factors by which the active site topology controls the activation of π-bonds. We specifically focus here on the CO molecule as CO dissociation plays a central role in processes such as Fischer–Tropsch synthesis (FTS)^21,22^ and CO_2_ methanation.^23^ For these processes, CO dissociation is not only a major rate-controlling step, but its barrier also determines the selectivity between CH_4_ and longer hydrocarbons in FTS^24^ and between CH_4_ and CO formation in CO_2_ methanation.^25^ Facile CO dissociation is observed over the transition metals Fe, Ru, Co, and Ni,^26^ for which nanoparticles can be supported on metal oxides like alumina, silica, titania, and magnesia.^26^ Depending on the active site, CO dissociation occurs either in a direct fashion or in an H-assisted manner via intermediates such as HCO, H_2_CO, or COH.

The C–O bond is very strong (1072 kJ/mol)^27^ and its scission requires the presence of a catalyst. The redistribution of electron density upon adsorption of CO on a transition metal destabilizes the CO triple-bond, providing access to a more facile dissociation pathway. Over the past few decades, many models have been constructed to describe this process. The most well-known model is from Blyholder^28^ who applied the theories of Orgel,^29,30^ Ballhausen,^31^ and Richardson^32^ about the bonding of a carbonyl as a ligand to a metal center to CO adsorption on extended transition metal surfaces. Using Hückel molecular orbital theory, Blyholder constructed a semiquantitative description on the nature of the metal-CO bond. The model predicts that electron donation from CO to the metal atom occurs by the interaction of the lone electron pair that resides on the C-terminus of the CO with the metal. This donation results in a large negative charge on the metal atom, giving rise to a backdonation from the metal to CO. This backdonation involves electrons from the *d*-orbitals of the metal, which are transferred to the antibonding π-orbitals of CO. Later, the term “Blyholder model” was used in a more general sense for models where only the frontier orbitals of CO, i.e., the HOMO and the LUMO, are involved in the bonding with the metal.^33^ According to this HOMO–LUMO model, CO chemisorption is the interaction of the 5σ orbital (HOMO) and 2π orbital (LUMO) with the *d*-orbitals of the metal. This interaction consists of CO donating electrons from the 5σ orbital to the metal *d*-band, called σ-donation, and the metal *d*-band donating electrons into 2π, referred to as π-backdonation. Within the HOMO–LUMO model, both σ-donation as well as π-backdonation are said to strengthen the metal-CO bond and weaken the C–O internal bond.

With advances in both computational resources and improved electronic structure models, several contributions were made to further refine upon the Blyholder model. Bagus et al.^34,35^ performed self-consistent-field calculations (SCF) for CO adsorbed on Na, Mg, and Al surfaces. They found that the electron donation from the 5σ orbital to the *d*-band is in fact very little and that the 5σ orbital is slightly antibonding for the metal-CO bond. This is because the metal σ-electrons move away from CO to reduce the Pauli repulsion with the electrons in the 5σ orbital. They state that the metal-CO bond mainly consists of electron donation from the metal into the 2π orbital.

Föhlisch et al.^36,37^ performed X-ray emission spectroscopy (XES) measurements in conjunction with density functional theory (DFT) calculations to understand CO bonding patterns on Cu and Ni surfaces. They found that after rehybridization of the 4σ and 5σ orbitals of CO with the d_σ_ orbitals of the metal, a σ-interaction exists that is repulsive for the metal-CO bond. This σ interaction, however, strengthens the internal C–O bond. As a counteracting effect, the 1π and 2π orbitals mix with the d_π_ orbitals of the metal and this π-interaction results in a weakening of the internal C–O bond and a strengthening of the metal-CO bond. The net result of these two counteracting effects determines the adsorption strength of CO and its activation. More recent DFT studies on CO adsorption on Ni and Cu surfaces are executed by Gameel et al.^38,39^ In their contributions, they unravel the role of the active site configuration and study the frontier molecular orbitals and charge redistribution. They suggest that the σ-interaction is indeed repulsive for Ni-CO, but it is partially repulsive and partially attractive for the Cu-CO bond. Furthermore, they found that C–O bond activation does not depend on the adsorption strength of CO but is strongly correlated with the coordination number of the metal–carbon interaction.

The previously mentioned studies focus primarily on one-, two-, three-, and four-fold adsorbed CO, yet it has been shown that five- and six-fold adsorbed CO gives rise to far lower CO dissociation barriers.^24^ Because both carbon and oxygen bind to the surface, orbital overlap is enhanced, potentially allowing for an increased electron transfer between CO and the metal. In a previous work, we showed that alloying Rh with Fe can result in a similar effect. The lower electronegativity of Fe gives rise to an enhanced charge transfer from the metal to CO, resulting in increased occupation of antibonding orbitals, leading to a reduced CO dissociation barrier in comparison to a pure Rh surface.^40^

In this contribution, we expand upon the previously constructed models for CO adsorption and bond activation by considering active site configurations allowing for five- and six-fold adsorbed CO. We studied electron redistribution and orbital hybridization by means of detailed density of states, crystal orbital Hamilton population, and DDEC6 charge analyses. The role of the σ- and π-systems in the bond (de)stabilization is explored and rationalized. We revisit the conclusions of the Blyholder model and place our observations into perspective with previous models developed in the open literature. The interpretation of the topology and local chemical environment of the active site toward modulating the dissociation barrier by the rearrangement of the molecular orbitals is crucial for the rational design of novel catalyst formulations.

## 2 Methods

### 2.1 DFT Calculations

Plane-wave density functional theory calculations were performed using the Vienna ab initio simulation package (VASP)^41,42^ that employs the projector-augmented wave (PAW) method to describe the core electrons.^43,44^ The Perdew–Burke–Ernzerhof (PBE) functional^45^ is used to describe electron exchange and correlation. PBE and its revised version by Hammer et al.^46^ (RPBE) were both considered. Note that no van der Waals corrections were applied, as after thorough testing we found that the calculations with and without van der Waals correction yielded similar results due to cancellation effects. An elaborate discussion can be found in Section S1 in the Supporting Information. Solutions to the Kohn–Sham equations were calculated using a plane wave basis set with a cutoff of 400 eV. For all calculations, spin polarization was included. The initial guess for the magnetic moment of each atom was set to 3.0 for the Co bulk, Co slab, and TiO_2_-supported Co models to ensure the systems converge to the magnetic ground state. For the Al_2_O_3_-supported Co models, the initial guess for the magnetic moment of each atom was set to 1.0, since for these systems, this value was sufficient for convergence to the magnetic ground state. We used the first-order Methfessel–Paxton method to apply smearing to the electrons, with a smearing width of 0.2 eV. Exception to this is the simulation of CO in the gas phase, for which we used Gaussian smearing with a smearing width of 5 × 10^–4^ eV. A discussion about the type of smearing and the smearing width can be found in Supporting Information Section S2. The Co FCC and HCP bulk phases were computed in unit cells of 3.51 × 3.51 × 3.51 Å and 2.49 × 2.49 × 4.02 Å, respectively. For both bulk cells, *k*-point convergence was reached with a mesh of 11 × 11 × 11 *k*-points (criterion of 1 meV/atom). For the extended surfaces, a *k*-point mesh of 5 × 5 x 1 is used to sample the Brillouin zone. The dimensions of the surface cells are 10.54 × 10.54 × 21.27 Å for Co(100), 9.94 × 10.54 × 22.21 Å for Co(110), 9.96 × 9.96 × 22.02 Å for Co(0001), and 8.64 × 9.47 × 21.55 Å for Co(112̅1). For the supported nanoclusters and -rods, a *k*-point mesh of 1 × 1 x 1, i.e., only the Γ-point, is used. The dimensions of these cells are 16.14 × 16.79 × 25.00 Å for the Co_55_/Al_2_O_3_ nanocluster and for the Co_84_/Al_2_O_3_ nanorod, and 17.74 × 19.55 × 26.49 Å for the Co_54_/TiO_2_ nanocluster and for the Co_81_/TiO_2_ nanorod. We optimized the stable states and the transition states using an ionic convergence criterion of 1 × 10^–4^ eV and an electronic convergence criterion of 1 × 10^–5^ eV. It was verified that all residual forces are less than 0.05 eV/Å for the adsorbate atoms in each Cartesian direction. All energies are corrected for the vibrational zero-point energy (ZPE). We obtained bulk Co–Co distances of 2.48 and 2.26 Å for FCC and HCP, respectively. These values are in good agreement with the reported experimental values of 2.51 Å^47^ and 2.29 Å for cobalt FCC and HCP, respectively.^48^ A more detailed discussion on the performance of the PBE XC-functional with respect to the cohesive energy of FCC and HCP Co is provided in Section S3. We searched for transition states with the nudged elastic band (NEB) method as implemented in VASP. We verified that the optimized transition states show one imaginary frequency in the direction of the reaction coordinate. For the optimized stable states, we verified that the frequencies are nonimaginary.

### 2.2 Model Systems

We placed Co(0001) (HCP), Co(112̅1) (HCP), Co(100) (FCC), and Co(110) (FCC) slabs at the center of the supercell. A vacuum slab of at least 15 Å was added to avoid spurious interactions between the adsorbates. The Co(0001) and Co(100) models consist of four layers, and the Co(112̅1) and Co(110) models consist of six layers. None of the layers in the slabs are frozen.

The supported nanoclusters and rods were created as follows. As supports for the nanoclusters and nanorods, we used γ-Al_2_O_3_(110) and rutile-TiO_2_(110) surfaces because these are reported to be thermodynamically the most stable.^8,49^ Four layers of the support material were placed in a supercell; the bottom two layers were frozen. After adding the nanocluster or nanorod, we enlarged the vacuum space above the slab to accommodate adsorbates, leaving a distance of at least 12 Å between neighboring super cells. The Co_55_/Al_2_O_3_ nanocluster model is based on the Ni_55_ cluster on the γ-Al_2_O_3_(110) surface of Silaghi et al.,^50^ where the Ni was replaced by Co. Starting from the hemispherical cobalt cluster of the Co_55_/Al_2_O_3_ model, three cobalt atoms were removed to create a pocket site with a B_5_ motif for the Co_52_/Al_2_O_3_ model. The Co_54_/TiO_2_ model was based on the latter but with two additional cobalt atoms at the base of the nanocluster to make it adhere to the TiO_2_ support. The continuous nanorods were built by starting with bare supports and adding one layer of cobalt atoms at a time, allowing the atoms to relax in between. On both Co_84_/Al_2_O_3_ and Co_81_/TiO_2_ nanorods, B_5_-like sites close to the metal–support interface were created by adding an extra layer of cobalt atoms on top of the existing nanorod. By covering the nanorod only partially with the extra layer, several stepped sites emerged. The stability of the nanoclusters and nanorods was assessed by calculating the energy corresponding to one Co atom detaching from the nanocluster and migrating to the Al_2_O_3_ or TiO_2_ support.^51^ A detailed discussion on the stability of the models can be found in Section S4 of the Supporting Information. Due to the use of sufficiently large vacuum slabs, no dipole corrections were applied. This is discussed in more detail in Section S5 of the Supporting Information.

### 2.3 DOS, COHP, and DDEC6 charges

Prior to electronic structure analysis, we performed an additional single-point calculations of the optimized pristine model systems and their corresponding states with CO adsorbed with VASP. The LOBSTER software^52−56^ was used to perform crystal orbital Hamilton population (COHP) and density of states (DOS) analyses. The number of bands in VASP and the number of local basis functions in LOBSTER were both set to the sum of the valence orbitals of all atoms present in the system. We used the pbeVaspFit2015 basis set^55,57,58^ with the basis functions [2s, 2p] for C and O, [3s, 3p] for Al, and [4s, 3p, 3d] for Ti and Co. For the DOS calculations, the basis functions were rotated in such a way that the *x*-axis is parallel to the C–O internal bond (using the “autorotate” keyword). For the COHP calculations, this was done for each atom–atom pair interaction to always align the bond axis with the global *x*-axis of the basis functions. We computed the COHP in an orbitalwise fashion. The absolute charge spilling was below 4.0% (average of the two spin channels) for all calculations. This means that at least 96% of the occupied wave function was projected onto the local basis functions. The absolute charge spilling could not be lowered by the employment of more basis functions. The Chargemol program version 3.5 was used to calculate the DDEC6 atomic charges.^59,60^

### 2.4 Research Data

Relevant input and output files for all calculations, necessary for facile reproduction of the results, are shared via a Zenodo repository.^61^ This repository also includes the set of Python scripts that have been used to parse the output files and produce the graphs.

## 3 Results and Discussion

### 3.1 Model Systems

**Figure 1:**
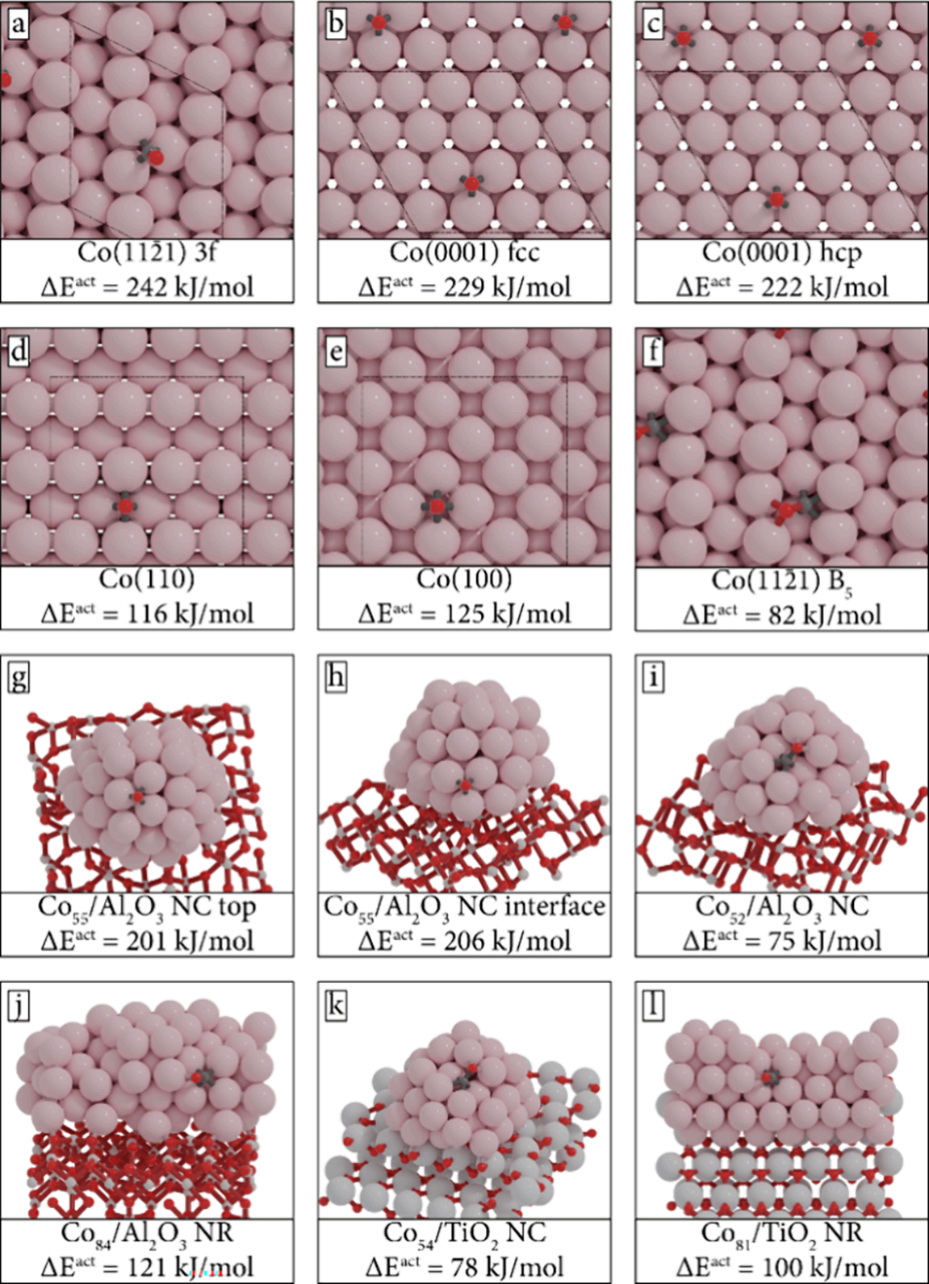
Geometry of the active site in the initial state of CO dissociation. NC and NR refer to nanoclusters and nanorods, respectively. Activation energies for direct CO dissociation are reported, including ZPE correction.

To unravel the electronic structure of CO bonding and activation on Co sites, a diverse set of model systems was studied including flat and stepped extended surfaces as well as nanoparticles and -rods supported on Al_2_O_3_ and TiO_2_. The set of model systems exposes a variety of active sites configurations, including planar 3-fold and 4-fold configurations, which give rise to one-, two-, three-, and four-fold CO adsorption, as well as B_5_ type step-edges, which can facilitate five- and six-fold CO adsorption. To investigate the influence of a metal–support interface, the supports TiO_2_ and Al_2_O_3_ are chosen to include both a reducible and a nonreducible metal oxide, respectively. In Figure 1, an overview is given of the 12 models, showing CO adsorbed in its predissociation state. Although some of our models provide top or bridge sites at which CO can easily adsorb, we discard top and bridge sites when studying CO dissociation. This is because CO would migrate from a top or bridge site to a three- or 4-fold site prior to the dissociation of the C–O bond in order to provide a stable transition state. It also offers a more stable final state for the C atom since C prefers a three- or four-fold coordination with Co over a top or bridge site. We initiated CO adsorption configurations different from the configurations shown in Figure 1, such as side-on adsorption on three- and four-fold sites and O-end adsorption. None of these attempts resulted in stable states.

In Figure 1a–f, six extended surface models are shown. Figure 1a displays the corrugated Co(112̅1) surface where CO adsorption occurs in a 3-fold mode within a B_5_ site. Figure 1b,c depict FCC and HCP sites on the closely packed Co(0001) surface, respectively. Figure 1d,e shows the (quasi-) 4-fold adsorption modes of CO on the open Co(110) and Co(100) surfaces, respectively. Lastly, Figure 1f pertains to a Co(112̅1) surface and displays a 6-fold adsorption. Herein, C is 4-fold coordinated to Co and O interacts with the two Co atoms at the upper edge of the B_5_ site. While the active site configurations on the extended surfaces as shown in Figure 1a–f have been thoroughly studied in the past, we repeated these calculations to establish a benchmark for comparing results obtained for the supported nanoclusters and -rods. A comparative analysis of our findings and the CO dissociation barriers that have been previously reported for these sites is presented in Table S9.

The supported nanoclusters and rods allow us to investigate active sites close to the metal–support interface. Herein, we vary the type of active site, its distance to the support interface, and also the type of the support. The nanoclusters are approximately 1 nm in diameter at the base. Both nanorods are continuous in one direction and represent the interfacial perimeter of the larger nanoparticles. Figure 1g–i shows the Al_2_O_3_-supported nanoclusters. In Figure 1g and h, CO is adsorbed in a 3-fold configuration either at the top of the nanoparticle, which is relatively far away from the interfacial perimeter (Figure 1g), or in a 3-fold site bordering the support (Figure 1h). The cobalt atoms of the 3-fold sites in Figure 1g,j have a lower coordination number than those found in Figure 1a–c. Figure 1i–l shows B_5_-like sites, similar to the B_5_ site in Figure 1f. Figure 1i,j is B_5_-like sites on an Al_2_O_3_-supported nanocluster and nanorod, respectively. Figure 1k,l show B_5_-like sites on a TiO_2_-supported nanocluster and nanorod, respectively.

### 3.2 CO Dissociation Pathways and Energetics

Here, we discuss the mode of CO adsorption, the CO dissociation pathway, and the corresponding reaction energetics for the 12 model systems as shown in Figure 1. The values reported are based on the PBE exchange-correlation functional. We compared these results with the RPBE exchange-correlation functional as shown in Section S6 in the Supporting Information, and we did not find any significant differences for the barriers. The reaction energetics and coordination numbers are provided in Table 1. The geometries of the transition and final states are shown in Figures S9 and S10, respectively. We discuss the geometry of CO dissociation on the 12 active sites elaborately in the Supporting Information in Section S7. Below, we discuss the two reaction steps on the Co(112̅1) surface, since we discuss the DOS and COHP of these steps in more detail in Section 3.4.

**Table 1:** CO Adsorption Energies and Forward and Backward Energies for CO Dissociation on the 12 Co Sites^a^

model	CO adsorption energy [kJ/mol]	CO dissociation barrier [kJ/mol]	C+O association energy [kJ/mol]	coordination of CO in initial state	coordination of CO in transition state
Co(112̅1) 3f	–166	242	130	C: 3; O: -	C: 3; O: 3
Co(0001) FCC	–158	229	110	C: 3; O: -	C: 3; O: 2
Co(0001) HCP	–160	222	135	C: 3; O: -	C: 3; O: 2
Co(110)	–135	116	105	C: 4; O: -	C: 4; O: 2
Co(100)	–175	125	168	C: 4; O: -	C: 4; O: 2
Co(112̅1) B_5_	–163	82	101	C: 4; O: 2	C: 4; O: 2
Co_55_/Al_2_O_3_ NC top	–169	201	165	C: 3; O: -	C: 3; O: 3
Co_55_/Al_2_O_3_ NC interface	–180	206	155	C: 3; O: -	C: 3; O: 2
Co_52_/Al_2_O_3_ NC	–152	75	80	C: 4; O: 2	C: 4; O: 2
Co_84_/Al_2_O_3_ NR	–156	121	138	C: 4; O: 1	C: 4; O: 2
Co_54_/TiO_2_ NC	–158	78	96	C: 4; O: 2	C: 4; O: 2
Co_81_/TiO_2_ NR	–152	100	111	C: 4; O: 1	C: 4; O: 3

a NC and NR refer to nanoclusters and nanorods, respectively. Activation energies for direct CO dissociation are reported including ZPE correction.

In Figure 1a, CO adsorbs on a 3-fold site as exposed on the Co(112̅1) surface. The adsorption energy is −166 kJ/mol. In the transition state, the oxygen moiety migrates to a neighboring active site and is bonded to the surface in a quasi-3-fold configuration. The carbon atom remains in the 3-fold site. In the transition state, C and O share two Co atoms. In the final state, the oxygen atom continues to migrate away from the carbon atom and adsorbs at an adjacent 3-fold site. Only a single Co atom is shared between C and O. This elementary reaction step has an activation energy of 242 kJ/mol and is endothermic by 112 kJ/mol.

Figure 1f displays the Co(112̅1) surface, which possesses a B_5_ site that accommodates a 6-fold adsorption of CO, with carbon and oxygen atoms bonding to the metal in a 4-fold and 2-fold configuration, respectively. Notably, carbon and oxygen atoms do not share any cobalt atoms in this initial state, and the adsorption energy is −163 kJ/mol. The transition state exhibits an elongated C–O bond due to the movement of O away from C while C remains stationary, with an activation energy of 82 kJ/mol. Subsequently, oxygen moves further between two cobalt atoms to bond with a third cobalt atom in the final state, where C and O atoms continue to not share any cobalt atoms. This CO dissociation process is slightly exothermic, with an energy release of 19 kJ/mol.

### 3.3 Approximating Orbital Overlap between CO and Co

The adsorption mode of CO and the proximity of CO to the Co atoms are correlated with the extent of orbital overlap. To understand how these factors affect the CO dissociation barrier, two metrics are introduced whose correlations with the dissociation barrier are studied. These two metrics correspond to the number of Co atoms the CO moiety is bonded to (CO coordination number) and the amount of overlap of probe electron density functions placed on CO and Co.

**Figure 2:**
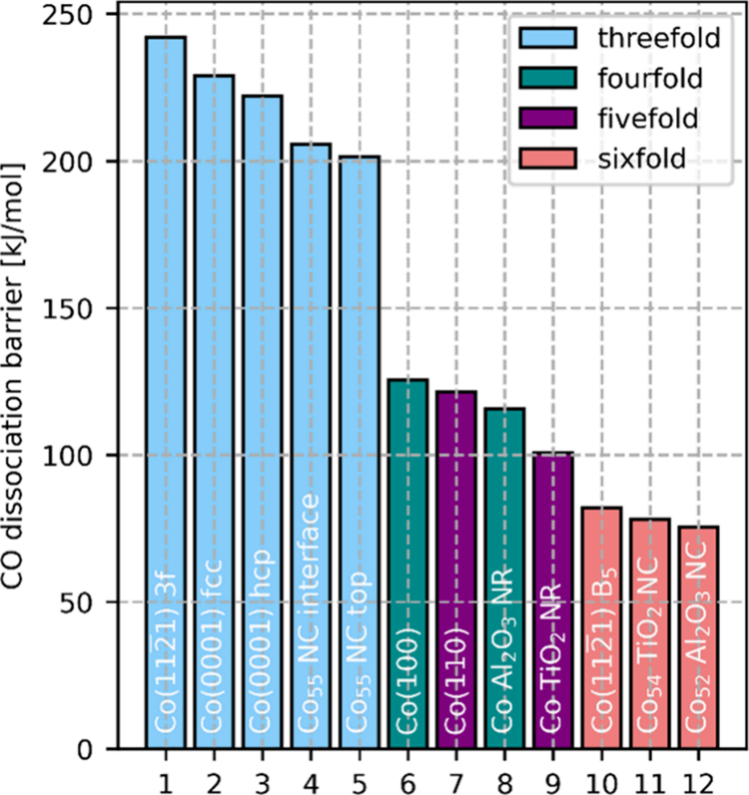
CO dissociation barriers ordered from high to low barrier, colored by the coordination of CO in the predissociation state.

To examine the coordination number dependency of the dissociation barriers, in Figure 2, the CO dissociation barriers for the various active sites are shown. The active sites are ordered from high to low dissociation barrier, and they are color-coded by the CO coordination number in the predissociation state. We consider two atoms to be bonded when the distance between them is less than 2.0 Å. From Figure 2, it is clear that a higher coordination of CO in the predissociation state coincides with a lower energy barrier for CO dissociation. This result is in line with Hammond’s postulate, which states that when the molecular structures of the predissociation state and the transition state resemble each other, the energies of these states will resemble each other as well.^62^ For five- and six-fold coordinated sites, O is already attached to Co. For these sites, the predissociation state of CO resembles the transition state more than for the three- and 4-fold coordinated sites, resulting in a lower reaction barrier.

Although the use of coordination numbers is an established procedure to describe chemical bonding,^63^ another approach was also considered. Rather than a predetermined cutoff radius that determines the coordination number, we considered the distance metric μ_1_, a sum of modified interatomic distances *d*_*ij*_ between the atoms as given by
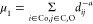
1wherein *i,j* loops over all the Co–C and Co–O distances and *p* (non-negative) is a power. Alternatively, we considered the overlap metric μ_2_, where we placed exponentially decaying functions on the Co, C, and O atoms and determined the overlap between these functions as given by
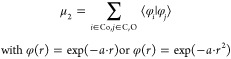
2wherein ϕ_*i*_(*r*), ϕ_*j*_(*r*) are functions centered at Co, C, or O. To find the best correlations, we optimized the fitting parameters α for μ_1_ and μ_2_. More details on the procedure can be found in Supporting Information Section S8.

**Figure 3:**
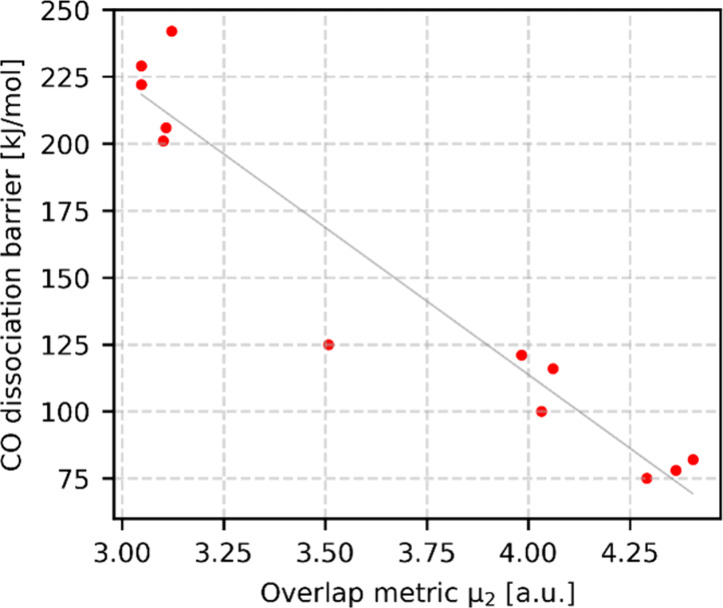
Correlation between CO dissociation barrier and the electron density overlap between CO and Co in the predissociation state. The overlap is computed as  where the electron density ϕ is modeled as Gaussian ϕ(*r*) = exp(−α · *r*^2^) with α = 0.97 for C, O, and α = 0.75 for Co. The Pearson correlation coefficient is −0.96.

Among all options considered, the best metric for the CO dissociation barrier corresponds to an overlap function μ_2_ (eq 2) wherein ϕ(*r*) = exp (−α · *r*^2^). This probe function mimics the electron density exponentially decaying with an increasing distance to the atom. The correlation between overlap and CO dissociation barrier is shown in Figure 3. The other correlations are shown in Figure S11. The Pearson correlation coefficient for the optimized correlation in Figure 3 is −0.96 and its coefficient of determination *R*^2^ is 0.92. This exponential function shows a better correlation with the CO dissociation barrier than the coordination number, even though it is also only based on the distance between the C, O, and Co atoms. This shows that the activation of CO is highly dependent on the distance between CO and the metal. The rationale is that the electron transfer between CO and the metal depends on the electron density overlap, which, in turn, depends on the distance. The degree of electron transfer determines the activation of the C–O bond.

### 3.4 Electronic Structure Analysis of CO Molecular Orbitals

To understand in more detail the mechanism of the bonding of CO to a cobalt site and its subsequent activation, an extensive electronic structure analysis using a *lm*-decomposed projected DOS (*lm*-pDOS) analysis, DDEC6 charge analysis, and COHP analysis is conducted. We will first present this analysis for two cases: for the 3-fold and 6-fold adsorption sites as exposed on the Co(112̅1) surface. These two situations have been chosen as they represent sufficiently distinct adsorption configurations and show a large difference in the CO dissociation barrier (242 and 82 kJ/mol, respectively). Thereafter, we generalize our observations for all model systems to establish correlations with the activation energy for CO dissociation.

#### 3.4.1 Density of States

**Figure 4:**
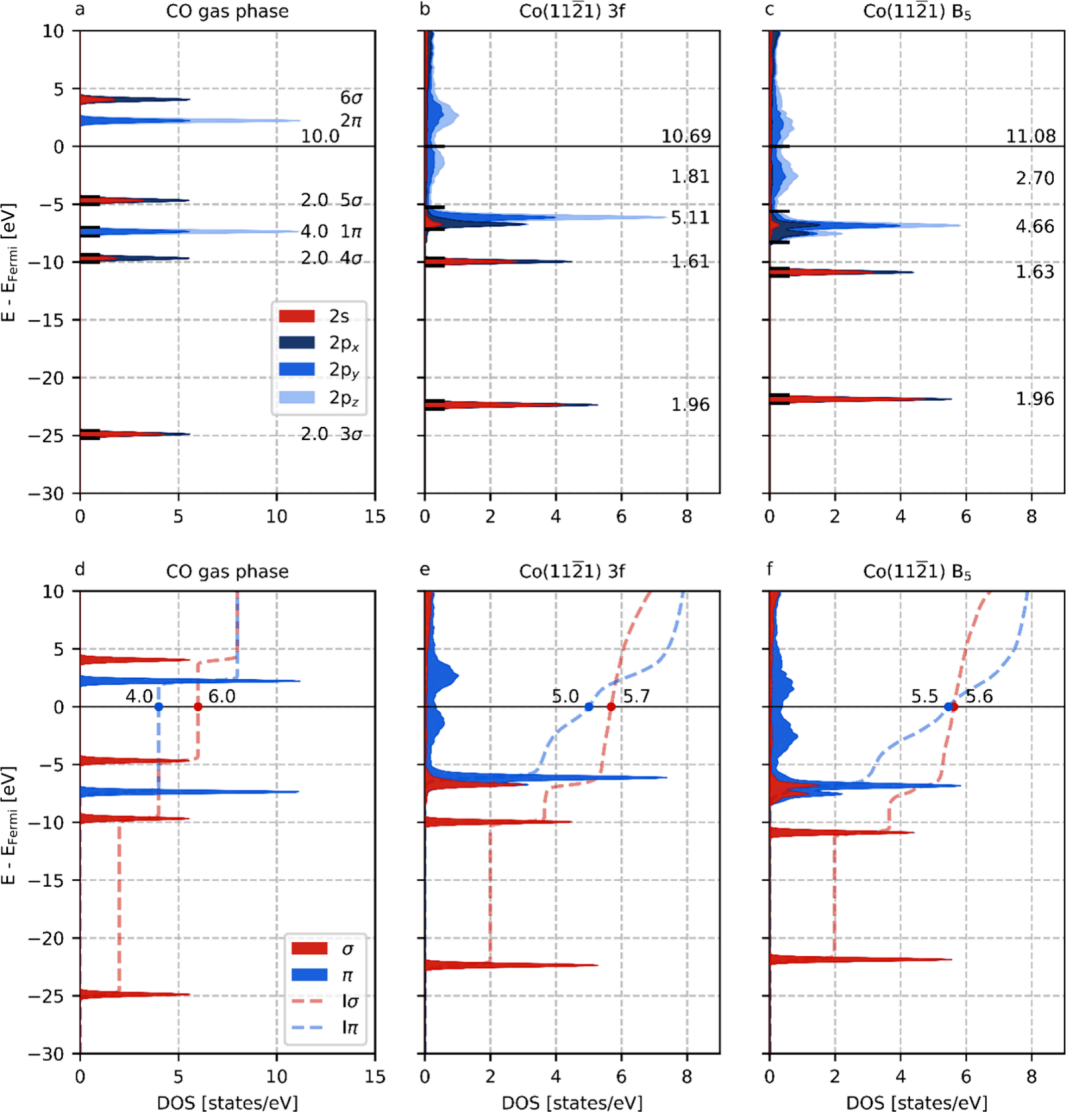
lm-pDOS analysis of CO gas phase (a,d), CO adsorbed on Co(112̅1) 3-fold site (b,e), and CO adsorbed on Co(112̅1) B_5_ (c,f). Subfigures a–c show the total and integrated DOS for CO, whereas subfigures d–f show the σ- and π-contributions. All energies are presented with respect to the Fermi-level. The IDOS at Fermi level is shown above the black line at zero energy (a–c) or near the red and blue dots (d–f). The values next to the peaks pertain to the area under the curves. The dashed lines show the integrated σ- and π-contributions (Iσ and Iπ).

In Figure 4, the DOS for CO adsorbed in the 3-fold (Figure 4b) and 6-fold (B_5_-site, Figure 4c) adsorption sites are shown. As a reference, in Figure 4a, the DOS of CO in the gas phase is included. All figures use the same reference energy, i.e., the zero of energy corresponding to the Fermi level of CO in the gas phase such that the peak positions can be readily compared. The molecular orbitals (MOs) are labeled based on their canonical names.^64^ In Figure 4a–c, the features are marked by horizontal black lines on opposite sides, and the area under the DOS curve is integrated to obtain the number of states per feature. In Figure 4d–f, the results obtained from Figure 4a–c are combined to generate separate DOS profiles for the σ-network, encompassing the 3σ, 4σ, and 5σ molecular orbitals, and the π-network, consisting of the 1π and 2π molecular orbitals. The sum of the number of electrons in the σ- and π-network corresponds to the integrated DOS (IDOS).

From Figure 4a–c, we observe that upon adsorption the number of electrons associated with CO increases from 10.0 (valence) electrons in the gas phase to 10.69 and 11.08 (valence) electrons for the 3-fold and 6-fold adsorbed configurations, respectively. The increase in electron density according to the DOS analysis is in good agreement with the charge of adsorbed CO according to the DDEC6 analysis, as shown in Figure S12. The number of electrons associated with adsorbed CO correlates well with the CO dissociation barrier, as shown in Figure S13. The 3σ and 4σ orbitals remain narrow upon adsorption, in line with their confined and localized nature due to their limited interaction with the metal *d*-band. Consequently, the corresponding peaks display subtle deviations from the gas phase situation. The 3σ peak undergoes an upward energy shift compared to the gas phase, indicative of increased electron–electron repulsion stemming from closer proximity to the surface. Similarly, the 1π peak also experiences an upward shift. In contrast, the 4σ, 5σ, and 2π peaks display a downward energy shift upon adsorption. This downward shift of the 2π orbital facilitates its partial filling, as it now resides below the Fermi level. Consequently, the adsorption-induced shift in the 2π orbital leads to its altered occupancy. Distinct from the behavior of the 3σ and 4σ orbitals, the 1π, 5σ, and formerly unoccupied 2π orbitals exhibit significant mixing with the Co *d*-states, resulting in peak broadening. The profoundness of this mixing is especially visible for the 1π and 5σ orbitals, whose states overlap in terms of energy. Differentiating between these two states is, however, possible by segregation of the σ- and π-contributions. As shown in Figure 4d–f, through the utilization of the *lm*-pDOS, we can attribute all 2s and 2p_*x*_ contributions to σ-bonding and thus the 5σ orbital, while the 2p_*y*_ and 2p_*z*_ contributions correspond to π-bonding and thus the 1π orbitals. From the same figures it can be readily observed that upon adsorption, the σ-system loses electrons with respect to the gas phase, whereas the π-system gains electrons. In the case of 3-fold adsorption, the number of electrons lost within the σ-system and gained within the π-system is comparatively lower than in the case of 6-fold adsorption. A movie (animation) of the changes in DOS and COHP plots upon adsorption of CO in 3-fold and 6-fold manner corresponding to Figures 4 and 6 can be found in the Supporting Information.

**Figure 5:**
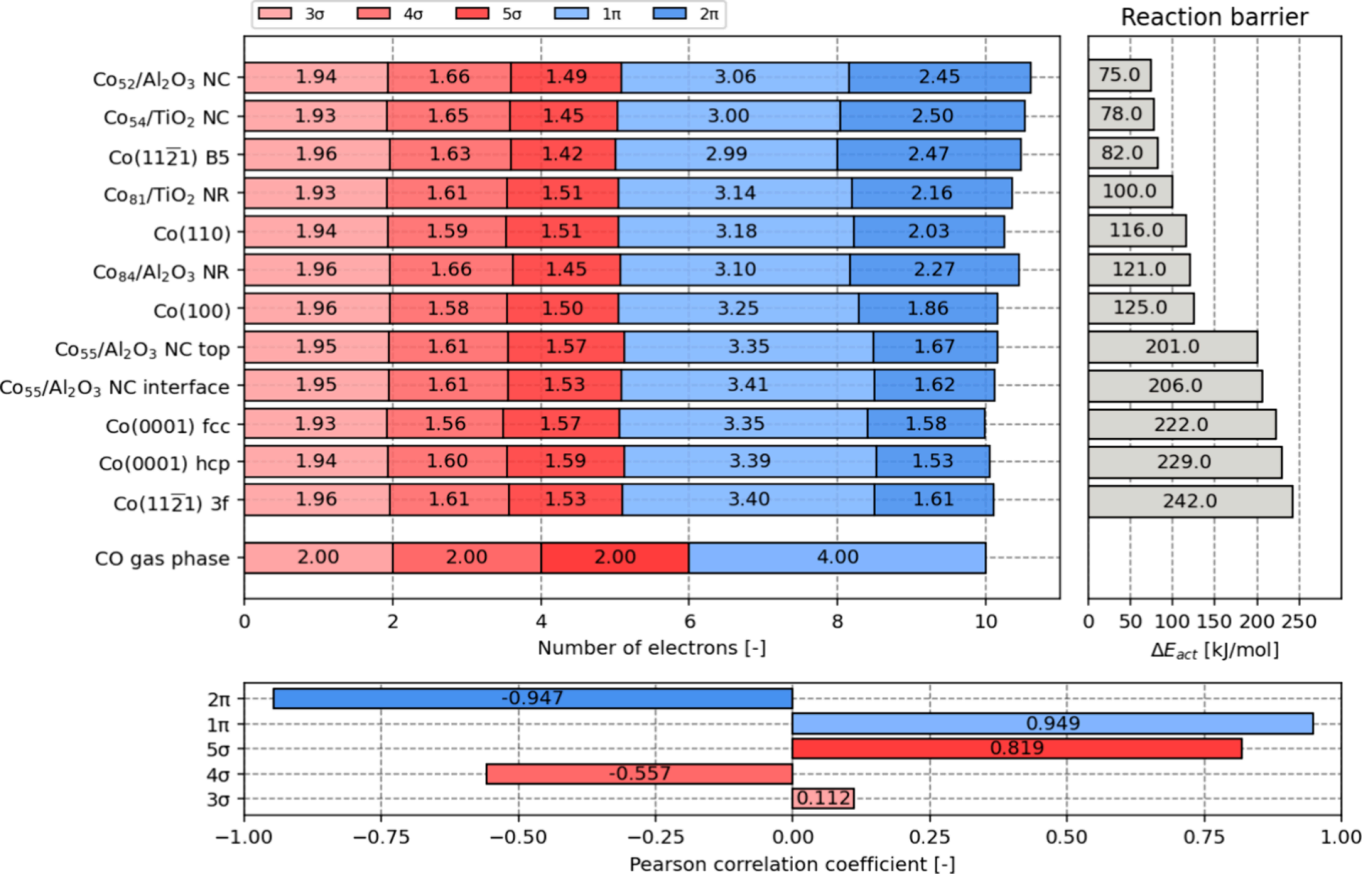
Molecular orbital DOS integrals for the various adsorption configurations of CO (upper left plot) and their corresponding reaction barriers (upper right plot). Pearson correlation coefficients for the correlation between the DOS integrals and the reaction barriers (lower plot).

The previous analysis is executed for all 12 model systems, and the results are collected in Figure 5. Based on the analysis presented in this figure, it becomes evident that the process of CO adsorption from the gas phase has a negligible impact on the number of electrons in the 3σ orbital, regardless of the specific adsorption mode or site under consideration. Regarding the 4σ and 5σ orbitals, it is observed that for each roughly half an electron is transferred from CO to the metal, irrespective of the adsorption mode.

**Figure 6:**
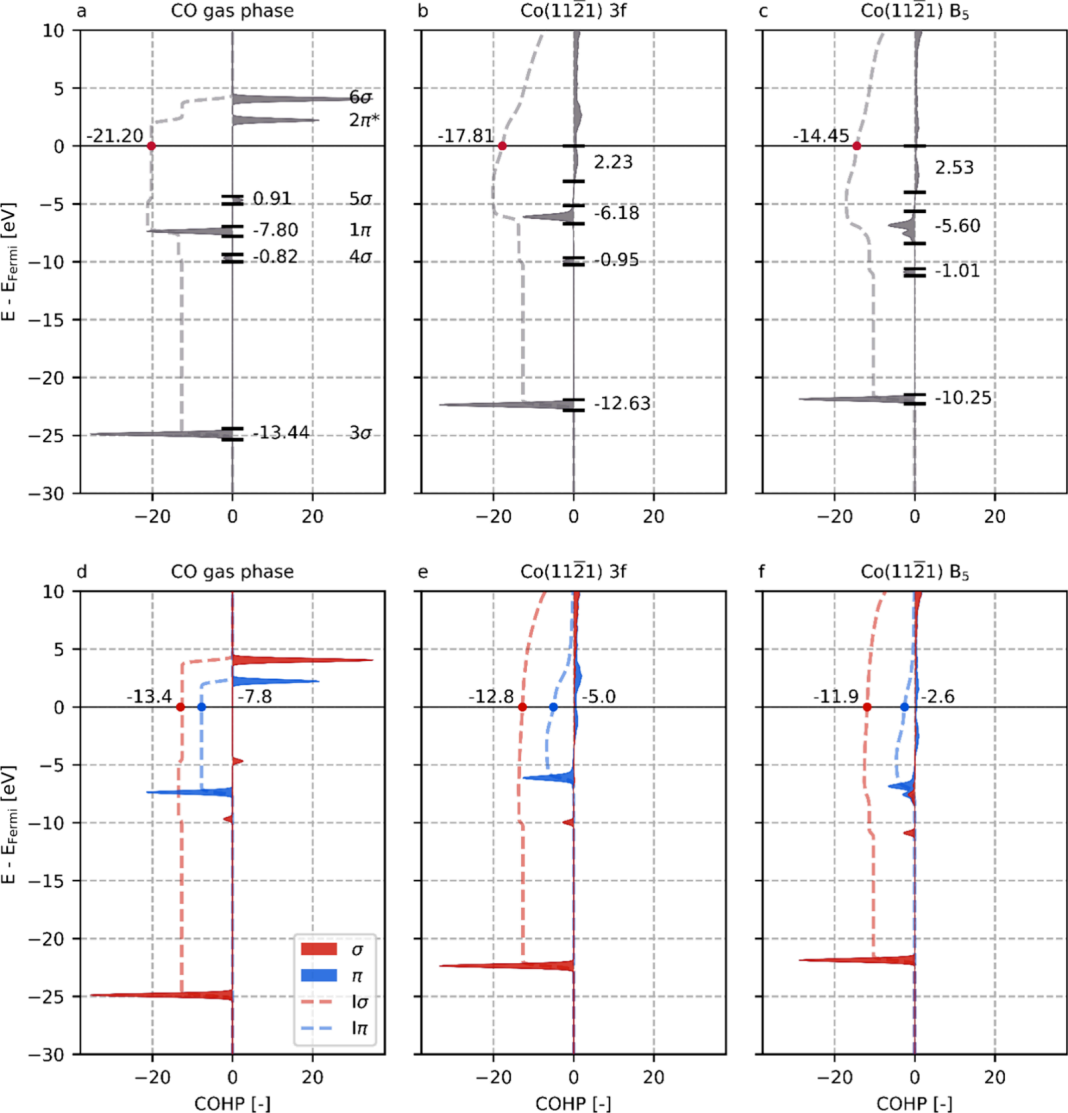
COHP as a function of the energy of the Kohn–Sham states of CO in the gas phase (a,d), CO adsorbed on Co(112̅1) 3f (b,e), and CO adsorbed on Co(112̅1) B_5_ (c,f). Subfigures a–c show the total and integrated COHP for CO, whereas subfigures d–f show the σ- and π-contributions. All plots use the same reference energy, corresponding to the Fermi level of CO in the gas phase. The ICOHP at the Fermi level is shown above the black line at zero energy (a–c) or near the red and blue dots (d–f). The values next to the peaks pertain to the area under the curves. The dashed lines show the integrated σ- and π-contributions (Iσ and Iπ).

The shifts in electron density within the 1π and 2π orbitals demonstrate a strong correlation with the dissociation barriers, as evidenced by Pearson correlation coefficients of 0.949 and −0.947, respectively. Conversely, the correlation between electron density in the 3σ, 4σ, and 5σ states and the dissociation barrier appears to be comparatively weaker, exhibiting Pearson correlation coefficients of −0.112, −0.567, and 0.819, respectively.

Hence, the quantification of electron loss from the 1π orbital of CO and the corresponding electron gain in the 2π orbital emerge as highly informative parameters to characterize the C–O dissociation barrier and, consequently, the extent of C–O activation. Remarkably, a substantial alteration in electron density within CO’s π-bondaccompanies CO activation, exhibiting a strong correlation. In contrast, a comparatively minor shift in electron density, less significantly correlated, is observed during CO activation within the σ-bond.

It is noteworthy that although the Pearson correlation coefficients and integrated DOS or COHP values are both quantitative measurements, combining the two to assess which MO modulation is most influential remains qualitative in nature, as the two individual measurements cannot be combined into a single meaningful quantitative model predicting dissociation barriers.

#### 3.4.2 COHP

The rearrangement of the electron density among the orbitals leads to destabilization of the C–O bond. The COHP method is an effective procedure to quantitatively assess this destabilization. By projection of the Kohn–Sham states onto local atomic orbitals, we can probe the interaction strength between any two atoms can be probed. The COHP analysis for gaseous and adsorbed CO on the Co(112̅1) 3f and B_5_ sites is visualized in Figure 6a–c. Akin to the procedure shown in Figure 4d–f, in Figure 6d–f, the COHP is split into σ- and π-contributions to distinguish between these networks.

In Figure 6a, we observe that for CO in the gas phase, the 3σ, 4σ, and 1π orbitals are bonding for the molecule, whereas the 5σ and unoccupied 2π orbitals are antibonding. For adsorbed CO in Figure 6b,c, it appears that the orbitals largely retain their bonding or antibonding character. While the DOS analysis indicates a minimal impact of the surface topology on the occupancy of the 3σ state, Figure 6 reveals a more pronounced influence on its corresponding integrated COHP (ICOHP) value. Despite the 3σ state exhibiting limited interference with the Co *d*-band preventing orbital mixing, the enhanced electron–electron repulsion arising from its closer proximity to the *d*-electrons leads to an electron redistribution such that the bonding character is severely diminished. Opposite to 3σ, the 4σ orbital increases in bonding character for C–O upon adsorption. This increase is small and rather constant for all adsorptions and, moreover, shows no correlation with the activation energy for C–O bond scission. Although the 4σ MO has a minor contribution to the C–O bond strength, it plays a large and consistent role in binding of CO to the Co site. From Figures S14 and S15, which show the COHP of the Co-CO bond, it can be seen that the 4σ MO contributes between approximately 25% to 35% to the total Co-CO bonding. Figure S16 shows a very small spread for the 4σ orbital; thus, this bonding contribution is constant for all adsorptions and thus independent of the adsorption mode.

Upon CO adsorption, the bonding character of the 1π orbital clearly decreases with respect to the value found for gas phase CO. The 5σ orbital, which is slightly antibonding in the gas phase, remains slightly antibonding for the 3f adsorption site. For the B_5_ adsorption site however, no σ-states are to be found in the vicinity of *E* = −5 eV. As already eluded upon in the DOS analysis, the strong mixing of the 5σ and 1π orbitals with the *d*-states of Co results in the formation of a set of new states with σ- and π-character. The availability of coordinatively unsaturated Co atoms in the B_5_ adsorption site leads to the formation of new stable states with σ-character that in contrast to the gas phase lie lower in energy as compared to the states with π-character. As a result, these states have a slight bonding character. Finally, the unoccupied 2π molecular orbitals in the gas phase descend below the Fermi level upon CO adsorption and thus become occupied. These states are antibonding irrespective of the adsorption site, though for the B_5_ site more electrons occupy these states, and hence these states exhibit a higher (more antibonding) COHP character.

**Figure 7:**
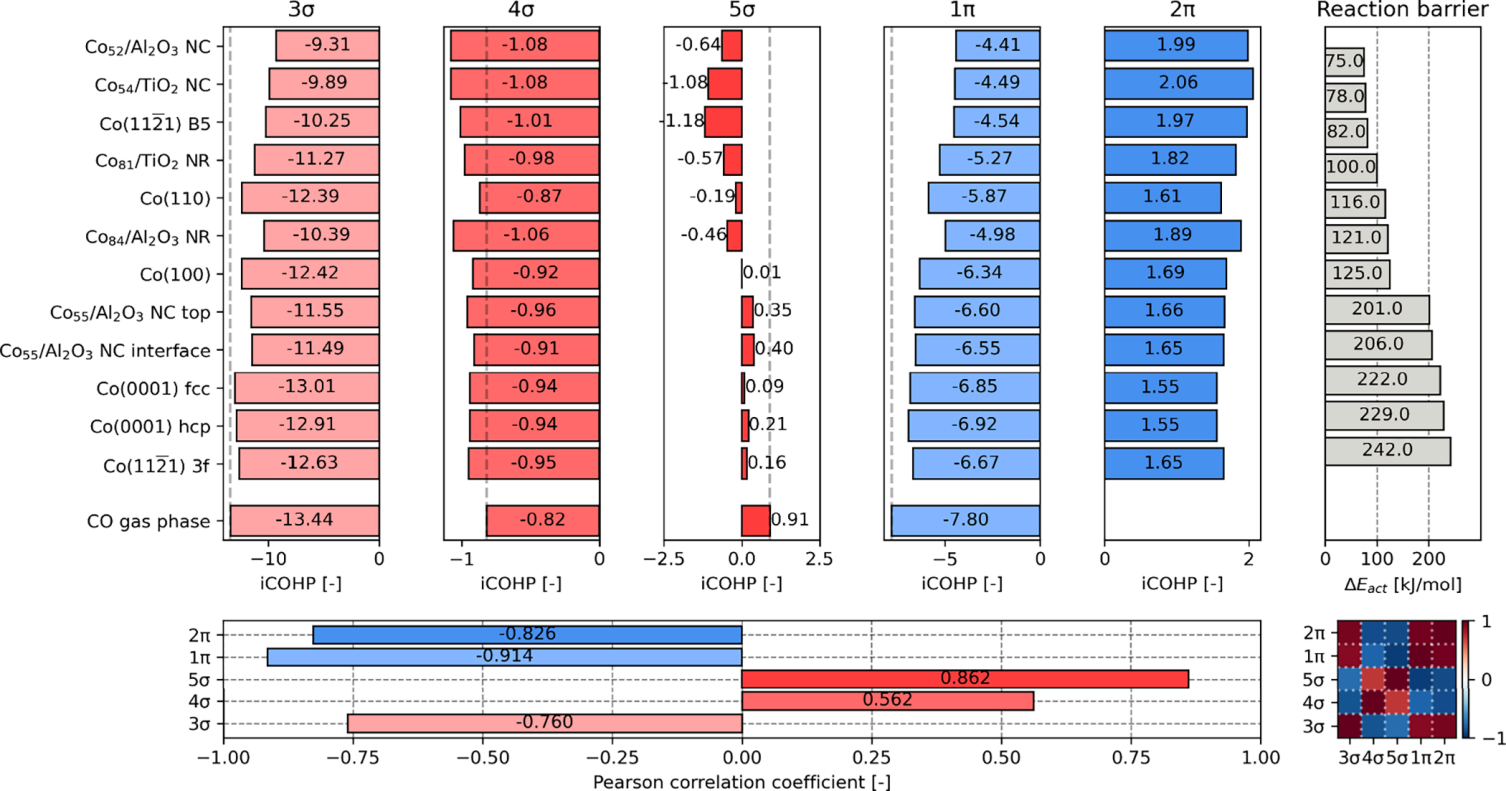
Molecular orbital COHP integrals for the various adsorption configurations of CO (five upper left plots) and their corresponding reaction barriers (upper right plot). Pearson correlation coefficients for the correlation between the COHP integrals and the reaction barriers (bottom left plot). Pearson correlation coefficients for correlations between the COHP integrals (bottom right plot).

Again, we can generalize these results for all of the systems that were studied. The collective data for all systems is visualized in Figure 7. We already established that the 3σ molecular orbital does not readily mix with *d*-states due to its compactness, although it increases in energy with respect to the Fermi level upon adsorption. This increase in energy is caused by electron–electron repulsion which is more pronounced the shorter the distance between C,O and the Co atoms (see also Figure 3). The COHP coefficients for the 3σ orbital clearly show this trend wherein a higher COHP value (less bonding) value is found as a function of decreasing C–O scission barrier. A Pearson correlation coefficient of −0.760 confirms this inverse trend, though this correlation should not be interpreted as the 3σ playing an important role in the bonding and activation of CO. It is rather that the COHP character of this molecular orbital serves as a proxy to characterize the proximity of CO with the metal surface. The 4σ orbital, whose electron distribution is somewhat more diffuse compared to the 3σ orbital, is less affected by the decrease in the distance between CO and the metal atoms. Consequently, we observe that its COHP character only marginally varies with changes in the site topology, as shown by a relatively poor Pearson correlation coefficient of 0.562. Stronger correlations are found for the 5σ and 1π orbitals, as indicated by their Pearson correlation coefficients of 0.862 and −0.914, respectively. These molecular orbitals strongly interact with the Co *d*-band. This interaction has a profound effect on their (anti)bonding character. For the 5σ molecular orbitals, it is observed that its COHP values decrease, i.e., that the MO becomes more bonding, with decreasing reaction barrier. Conversely, for the 1π orbital, it is seen that its COHP value increases, thus becoming less bonding with decreasing reaction barrier. The variations in the character of the COHP for the 1π and 2π orbitals can be rationalized based on the electron occupancy assigned to these states. Considering the presence of a nodal plane along the bonding axis, the 1π atomic orbitals inherently possess bonding character. The reduction in the integrated COHP pertaining to these states merely reflects their diminished occupancy, as depicted in Figure 5. A parallel rationale applies to the 2π states, which exhibit not only a nodal plane along the bonding axis but also a perpendicular plane intersecting the C–O bond. Consequently, these states inherently manifest antibonding character. When more electrons are donated into these states, simultaneously, the overall integrated COHP increases and the reaction barrier decreases.

**Figure 8:**
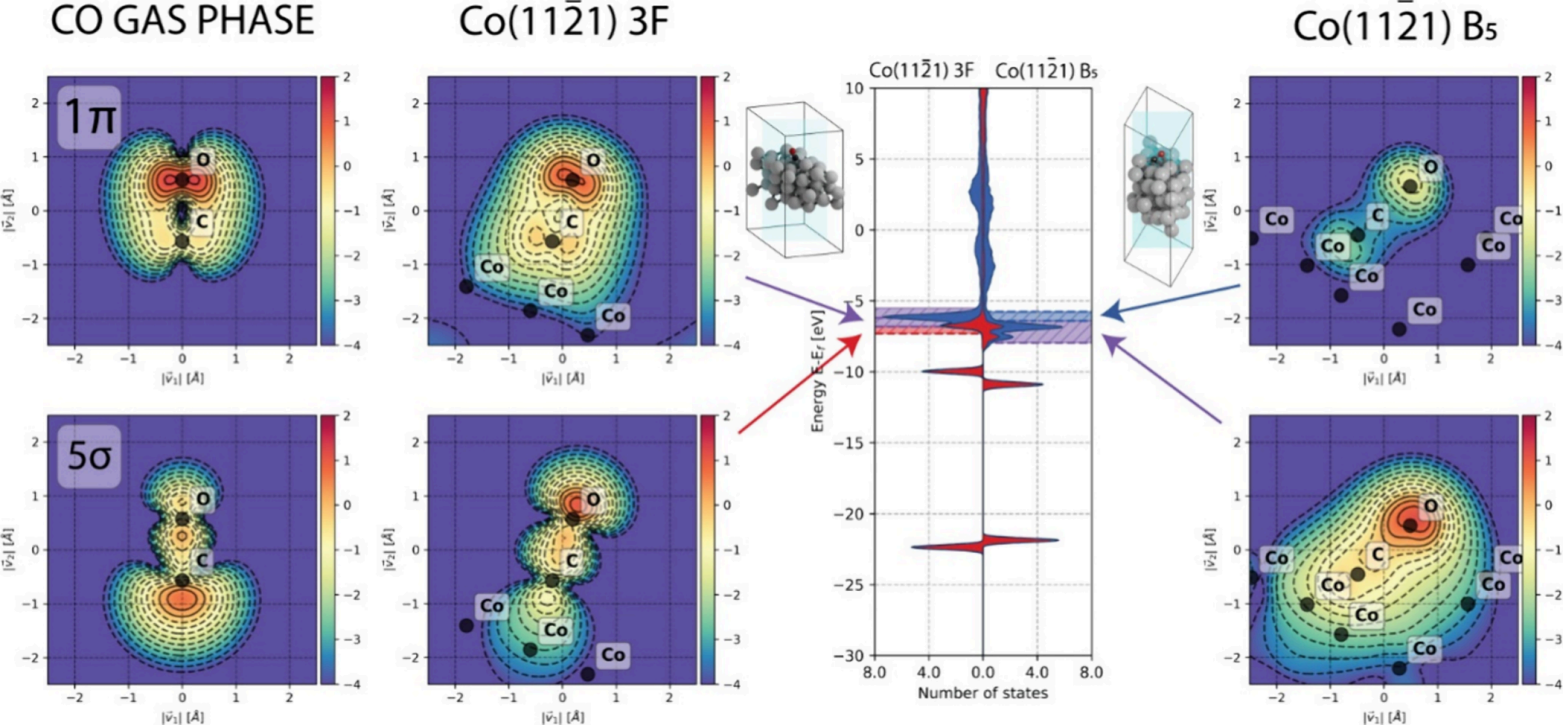
Electron density plots corresponding to characteristic energy regions of the DOS for the Co(112̅1) 3f system (center two contour plots) and Co(112̅1) B_5_ system (right two contour plots). In the DOS graph, red shows the σ- and blue shows the π-contribution. For comparison purposes, the electron density associated with the 5σ and 1π molecular orbitals for gaseous CO are shown as well (leftmost two contour plots). The characteristic energy regions are shown by the hatched rectangles in the DOS graph. On opposite sides of the DOS graph, a schematic depiction is provided how the contour plane is oriented with respect to the unit cell.

For the 5σ orbital, a change from antibonding to bonding is observed upon a decrease of the reaction barrier, indicating that the bonding character of the 5σ orbital shows an opposite trend with respect to the overall strength of the bond. To understand this behavior, we need to consider the electron density associated with the 5σ bond, which is hindered by the fact that the 5σ and 1π states overlap in energy. In Figure 8, contour plots of the electron density corresponding to the region of interest for the Co(112̅1) 3f and Co(112̅1) B_5_ systems are shown. For reference, the contour plots for the 5σ and 1π molecular orbitals in gaseous CO are also shown. The energy intervals used to construct these contour plots are indicated by the hashed rectangles in the DOS graph in the center of the figure. These energy intervals have been chosen such that they allow for qualitative analysis.

We can readily observe that the contour plots of the 5σ states and 1π regions for Co(112̅1) 3f show striking similarity. Upon adsorption, the electron density in the 5σ MO shifts from its predominant presence around the carbon atom to the oxygen atom. This shift alleviates the unfavorable electron–electron repulsion with an increase in the electron density on the cobalt surface. Despite this redistribution of electron density, we can observe the preservation of two nodal planes perpendicular to the bonding axis upon adsorption. Consequently, this preservation results in a minor antibonding molecular orbital, akin to the situation in the gas phase, consistent with the COHP values. Similarly, the nodal characteristics of the 1π molecular orbital remain intact after adsorption, leading to an overall bonding character.

The analysis of the Co(112̅1) B_5_ system is more intricate due to the overlapping nature of the 5σ and 1π states. In the region where these states overlap, the electron density reveals a slight decrease in electron density close to the C atom and similar to the Co(112̅1) 3f system a migration of electron density to the O atom. Our interpretation of the situation is that when compared to the Co(112̅1) 3f system, the 5σ states exhibit a reduced antibonding character, primarily attributed to the disappearance of the two nodal planes perpendicular to the bonding axis.

Given the similar features observed in the DOS for both the σ- and π-systems in this region of interest, we infer that the electron density as shown in the contour plot is also characteristic of the 1π states. For the 1π molecular orbital in the gas phase as well as the 1π states for the Co(112̅1) 3f system, we observe that the electron density is almost symmetrically distributed around the C–O bonding axis, resulting in a favorable interaction as evidenced by the negative COHP values. In contrast, due to the close proximity of the CO molecule with respect to the catalytic surface for the Co(112̅1) B_5_ system, the electron density is redistributed to mitigate unfavorable electron–electron repulsion. Consequently, the electron density resides predominantly on the opposite side of the C–O bond with respect to the surface. This rather asymmetrical electron density distribution is less favorable for the C–O bonding, and thus an increase in the COHP value (i.e., more antibonding) is observed for the 1π states in the Co(112̅1) B_5_ system as compared to the Co(112̅1) 3f system or the gas phase.

Based on the results discussed, we are now able to perform a qualitative assessment of which changes in shape and occupation of the canonical molecular orbitals have the largest influence on the dissociation barrier. Note that for this assessment, we cannot solely rely on using the Pearson correlation coefficients as these only measure the extent of linear correlation between the samples and not the relative impacts of the different orbitals on the CO dissociation barrier (i.e., the slopes). The slope of the linear relationship can be readily assessed by considering the absolute changes in the iCOHP values. Here, we find that the largest changes in iCOHP for the MOs that show a negative correlation with the reaction barrier are ranked as 3σ > 1π > 2π. Since the changes for 3σ show a somewhat weaker correlation with the barriers than 1π (Pearson correlation coefficient of 3σ is 0.760), we conclude that changes to the 1π orbital (Pearson correlation coefficient of 0.914) are most influential in lowering the CO dissociation barrier, closely followed by changes in 3σ and to a lesser extent by the occupation of the 2π MO.

### 3.5 Comparison with Literature Models

The original paper from Blyholder^28^ primarily focuses on π-backdonation from the metal to CO, resulting in a weakening of the C–O bond. Similar to these results, we find a net transfer of electron density from the metal to CO. The formerly empty 2π-orbitals receive electron density from Co. In contrast, the 1π orbitals donate electron density to the metal. Electron donation from the 1π-orbitals and back-donation to the 2π-orbitals both strengthen the M-CO bond while weakening the internal C–O bond. Like the Blyholder model, we find that the σ interactions play a less dominant role.

Föhlisch et al.^36,37^ proposed a chemisorption model for Cu-CO and Ni-CO where the π- and σ-interactions have opposed effects. The former interaction strengthens the metal–CO bond while weakening the internal C–O bond, whereas the latter does the opposite. For the π interaction, our findings are in line with this result. However, for the σ-interaction, we do not find a counteracting effect, yet predict it has a similar effect asthe π-interaction, yet to a lesser extent. Although the electron redistribution in the highest lying σ-orbitals, i.e., the 4σ and 5σ MOs, results in a strengthening of the C–O bond upon adsorption, the C–O destabilization caused by the 3σ MO is of a greater magnitude.

More recently, Gameel et al.^38^ examined the electronic structure of CO adsorbed on Cu, and later also on Ni.^39^ They draw similar conclusions for CO on Ni and Cu as we do for Co. When metal-CO coordination increases, more electron density is transferred to the 2π orbitals and the C–O bond is weakened. Similar to our findings, they did not observe a correlation between the CO adsorption energy and C–O bond activation. The authors examine the 3σ and 1π orbitals in detail, rationalizing orbital destabilization based on the increase in energy of the eigenvalues of the Kohn–Sham states and broadening of the density of states. Our results for CO on Co show the same upshift for 3σ and a broadening of 1π. Gameel et al. also conclude that the broadening of 1π has a larger effect on C–O bond destabilization than the alterations in the 3σ orbital. Our observation that the electron loss in the 1π orbitals and the electron redistribution in the 3σ orbital are the most important factors for C–O bond weakening and are in agreement with their results. We thus conclude that a high degree of similarity exists for CO activation for these three late transition metals.

## 4 Conclusions

We investigated the electronic structure of CO adsorbed on various Co sites, which displayed vast differences in the CO dissociation barrier. Geometric analysis of the adsorbate-site topology reveals that the electron density overlap between M-C and M-O acts as an accurate descriptor for the CO dissociation barrier. To understand the underlying electronic effects of this observation, detailed density of states, crystal orbital Hamilton population, and DDEC6 charge analyses were conducted to rationalize the changes in activation energies based on orbital hybridization and charge transfer.

For each of the canonical molecular orbitals in CO, we identified the trends between weakly and strongly activating active site configurations based on their charge and bonding characteristics. We found that the 3σ orbital retains its total charge upon adsorption; however, the electron density redistributes to reduce electron–electron repulsion with the *d*-band. This results in a blueshift of the 3σ orbital, weakening the C–O bond.

The 4σ and 5σ orbitals both lose a constant amount of electron density upon adsorption, independent of the adsorption mode and corresponding to a total of about 0.9 electrons with respect to the gas phase. This loss in electron density and the redistribution of the electron density of these orbitals result for both orbitals in a strengthening of the C–O bond. This effect is rather small for the 4σ molecular orbital yet more pronounced for the 5σ orbital. For 3-fold and 4-fold adsorption modes, we attribute the small increase in bond strength of 5σ to the migration of electron density from C toward the O terminus, leading to a loss of nodal plane character perpendicular to the C–O bonding axis present in the 5σ orbital of gaseous CO. For five- and six-fold adsorption modes, the increase in bond strength is larger because these perpendicular nodal planes disappear altogether, manifesting in an overall bonding characteristic of the 5σ orbital for these configurations.

For the 1π orbital, it is found that a significant amount of electron density is donated to Co upon adsorption, a feature that is strongly correlated to the CO dissociation barrier. Like the 5σ orbital, we assign this observation to the changes that occur in the nodal planes. For three- and 4-fold adsorption modes, the 1π molecular orbital retains its nodal plane alongside the C–O bonding axis and thus its bonding character. Therefore, the loss of electron density in the 1π orbital leads to a weakening of the C–O bond. For five- and six-fold adsorption modes, the enhanced electron–electron repulsion results in a further distancing of the 1π orbital with respect to the Co atoms, leading to a shift of the 1π nodal plane away from the Co surface. This weakens the C–O bond. The 2π orbital, which is unoccupied for CO in the gas phase, gains up to 2.5 electrons upon adsorption. Both the increase in electron density and the increase in antibonding character of the 2π orbital portray strong correlations with the CO dissociation barrier.

When we distinguish between σ- and π-systems, we observe that, in total, both systems are strengthening the Co-CO bond and weakening the C–O bond upon adsorption. The individual components of the σ-system play different roles. 4σ shows a constant C–O bond strengthening independent of the adsorption mode, while 5σ contributes more to strengthening the C–O bond upon more activated adsorption. The 3σ bond weakens the C–O bond upon adsorption and is more weak for more activated adsorptions. The π-system has a critical role in the activation of CO, with both 1π and 2π largely contributing to this. Both the electron donation from 1π to the *d*-band and the backdonation into 2π become more pronounced upon more activated adsorption.

In this article, we provided an electronic structure level understanding of how geometrical and charge-transfer factors modulate the CO dissociation barrier. We identified changes to the 3σ and 1π molecular orbitals to be most influential in affecting the barrier, a process that can be induced by facilitating an active site configuration that allows for a tilted CO adsorption such as a B_5_ motif. This understanding can inspire new experimental avenues toward novel catalyst nanoparticle formulations exposing specific highly active site configurations leading to more active and selective catalyst materials.

## Supporting Information Available

Section S1: comparison with and without van der Waals correction; Table S1: CO adsorption and CO dissociation energies on Co(0001) HCP site with and without van der Waals correction; Table S2: CO adsorption and CO dissociation energies on the Co(112̅1) B_5_ site with and without van der Waals correction; Table S3: CO adsorption and CO dissociation energies on Co_52_/Al_2_O_3_ NC site with and without van der Waals correction; Table S4: CO adsorption and CO dissociation energies on Co_81_/TiO_2_ NR site with and without van der Waals correction; Figure S1: DOS plots of predissociation state of CO on Co(0001) HCP site with and without van der Waals correction; Figure S2: COHP plots of predissociation state of CO on the Co(0001) HCP site with and without van der Waals correction; Figure S3: DOS plots of predissociation state of CO on the Co(112̅1) B_5_ site with and without van der Waals correction; Figure S4: COHP plots of predissociation state of CO on Co(112̅1) B_5_ site with and without van der Waals correction; Figure S5: DOS plots of predissociation state of CO on the Co_52_/Al_2_O_3_ NC site with and without van der Waals correction; Figure S6: COHP plots of predissociation state of the CO on Co_52_/Al_2_O_3_ NC site with and without van der Waals correction; Figure S7: DOS plots of predissociation state of the CO on Co_81_/TiO_2_ NR site with and without van der Waals correction. Figure S8: COHP plots of predissociation state of CO on Co_81_/TiO_2_ NR site with and without van der Waals correction. Section S2: Type of smearing and smearing width; Table S5: absolute energy difference between different smearing settings; Section S3: performance of PBE on the cohesive energy of FCC and HCP Co; Section S4: assessment of stability of supported Co nanoclusters and nanorods; Table S6: Co detachment energy of supported Co nanoclusters and nanorods; Section S5: dipole correction; Table S7: absolute energy difference between calculations with and without dipole correction; Section S6: comparison between PBE and RPBE; Table S8: comparison of CO adsorption energies, forward and backward energies for CO dissociation between PBE and revised PBE from Hammer et al.; Figure S9: geometry of transition state of CO dissociation on 12 Co sites; Figure S10: geometry of final state of CO dissociation on 12 Co sites; Section S7: description of initial, transition, and final states of CO dissociation; Table S9: forward and backward CO dissociation energies for the extended surface models and the values reported in the literature; Section S8: approximating orbital overlap between CO and Co; Figure S11: correlation between CO dissociation barrier and the electron density overlap between CO and Co in the predissociation state; Figure S12: correlation between IDOS at Fermi level and the DDEC6 charge of adsorbed CO. Figure S13: correlation between CO dissociation barrier and IDOS at the Fermi level; Figure S14: COHP as function of the energy of the Kohn–Sham states of the Co-CO bond for CO adsorbed on Co(112̅1) 3f and CO adsorbed on Co(112̅1) B_5_; Figure S15: σ- and π-contributions of the COHP as function of the energy of the Kohn–Sham states of the Co-CO bond for CO adsorbed on Co(112̅1) 3f and CO adsorbed on Co(112̅1) B_5_; Figure S16: boxplot of the COHP values of the Co-CO interactions (PDF)
Movie (animation) of the changes in DOS and COHP plots upon adsorption of CO in 3-fold and 6-fold manner corresponding to Figures 4 and 6 (MP4, MP4)
