# Supplementary material for: Unravelling CO Activation on Flat and Stepped Co Surfaces: A Molecular Orbital Analysis
Source: J Phys Chem C Nanomater Interfaces. 2024 May 23;128(22):8947–60. doi: 10.1021/acs.jpcc.4c00144 (PMC11163463; doi:10.1021/acs.jpcc.4c00144)
Supplement: Supplementary file 1 — jp4c00144_si_001.pdf [file jp4c00144_si_001.pdf]

**Supporting Information**

# Unravelling CO Activation on Flat and Stepped Co Surfaces: A Molecular Orbital Analysis

Rozemarijn D.E. Krösschell, Emiel J.M. Hensen, Ivo A.W. Filot*

Laboratory of Inorganic Materials & Catalysis, Department of Chemical Engineering and Chemistry, Eindhoven University of Technology, PO Box 513, 5600 MB, Eindhoven, The Netherlands.

*i.a.w.filot@tue.nl

**Table of Contents**

Section S1: *Comparison with and without Van der Waals correction.*

Table S1: *CO adsorption and CO dissociation energies on Co(0001) HCP site with and without Van der Waals correction.*

Table S2: *CO adsorption and CO dissociation energies on Co(11$\bar{2}$1) $B_5$ site with and without Van der Waals correction.*

Table S3: *CO adsorption and CO dissociation energies on $Co_{52}/Al_2O_3$ NC site with and without Van der Waals correction.*

Table S4: *CO adsorption and CO dissociation energies on $Co_{81}/TiO_2$ NR site with and without Van der Waals correction.*

Figure S1: *DOS plots of predissociation state of CO on Co(0001) HCP site with and without Van der Waals correction.*

Figure S2: *COHP plots of predissociation state of CO on Co(0001) HCP site with and without Van der Waals correction.*

Figure S3: *DOS plots of predissociation state of CO on Co(11$\bar{2}$1) $B_5$ site with and without Van der Waals correction.*

Figure S4: *COHP plots of predissociation state of CO on Co(11$\bar{2}$1) $B_5$ site with and without Van der Waals correction.*

Figure S5: *DOS plots of predissociation state of CO on $Co_{52}/Al_2O_3$ NC site with and without Van der Waals correction.*

Figure S6: *COHP plots of predissociation state of CO on $Co_{52}/Al_2O_3$ NC site with and without Van der Waals correction.*

Figure S7: *DOS plots of predissociation state of CO on $Co_{81}/TiO_2$ NR site with and without Van der Waals correction.*

Figure S8: *COHP plots of predissociation state of CO on $Co_{81}/TiO_2$ NR site with and without Van der Waals correction.*

Section S2: *Type of smearing and smearing width.*

Table S5: *Absolute energy difference between different smearing settings.*

Section S3: *Performance of PBE on the cohesive energy of FCC and HCP Co.*

Section S4: *Assessment of stability of supported Co nanoclusters and nanorods.*

Table S6: *Co detachment energy of supported Co nanoclusters and nanorods.*

Section S5: *Dipole correction.*

Table S7: *Absolute energy difference between calculations with and without dipole correction.*

Section S6: *Comparison between PBE and RPBE.*

Table S8: *Comparison of CO adsorption energies, forward and backward energies for CO dissociation between PBE and revised PBE from Hammer et al.*

Figure S9: *Geometry of transition state of CO dissociation on twelve Co sites.*

Figure S10: *Geometry of final state of CO dissociation on twelve Co sites.*

Section S7: *Description of initial, transition and final states of CO dissociation.*

Table S9: *Forward and backward CO dissociation energies for the extended surface models and the values reported in the literature.*

Section S8: *Approximating orbital overlap between CO and Co.*

Figure S11: *Correlation between CO dissociation barrier and the electron density overlap between CO and Co in the predissociation state.*

Figure S12: *Correlation between IDOS at Fermi level and the DDEC6 charge of adsorbed CO.*

Figure S13: *Correlation between CO dissociation barrier and IDOS at Fermi level.*

Figure S14: *COHP as function of the energy of the Kohn-Sham states of the Co-CO bond for CO adsorbed on Co(11$\bar{2}$1) 3f (a,c), and CO adsorbed on Co(11$\bar{2}$1) $B_5$ (b,d).*

Figure S15: *σ- and π-contributions of the COHP as function of the energy of the Kohn-Sham states of the Co-CO bond for CO adsorbed on Co(11$\bar{2}$1) 3f (a), and CO adsorbed on Co(11$\bar{2}$1) $B_5$ (b).*

Figure S16: *Boxplot of the COHP values of the Co-CO interactions.*

References

**Section S1**: *Comparison with and without Van der Waals correction.*

For four sites, we compare the electronic density of CO adsorption and dissociation over that site with no Van der Waals correction, the Van der Waals correction DFT-D3 method of Grimme with zero-damping function[1] and the Van der Waals correction DFT-D3 method of Grimme with Becke-Johnson damping function.[2] To apply no Van der Waals correction in VASP, one specifies IVDW = 0 for no Van der Waals correction, IVDW = 11 for the Van der Waals correction DFT-D3 method of Grimme with zero-damping function and IVDW = 12 for the Van der Waals correction DFT-D3 method of Grimme with Becke-Johnson damping function. For the sake of brevity, the labels IVDW0, IVDW11 and IVDW12, respectively, are used in this Section to distinguish between these three cases.

The CO adsorption and CO dissociation energies are compared for the different Van der Waals corrections in Tables S1-S4 for four representative active sites. The DOS and COHP plots that correspond to CO in predissociation state on these sites are shown in Figures S5-S12. On top of the qualitative comparison of the DOS and COHP plots, we have computed the average absolute deviation per data point for DOS and COHP with respect to the calculation that has no Van der Waals correction applied. The average absolute deviation per data point is calculated for the data points up to Fermi level. These values are listed in the Tables S1-S4 in the last two columns.

**Table S1**: *CO adsorption and CO dissociation energies on Co(0001) HCP site with and without Van der Waals correction.*

| Co(0001) HCP site | | | | | |
|---|---|---|---|---|---|
| Van der Waals correction | CO adsorption energy [kJ/mol] | CO dissociation energy forward [kJ/mol] | CO dissociation energy backward [kJ/mol] | DOS average absolute deviation per data point | COHP average absolute deviation per data point |
| IVDW0 | -163 | 226 | 140 | 0.00E+00 | 0.00E+00 |
| IVDW11 | -183 | 222 | 143 | 8.79E-03 | 1.92E-02 |
| IVDW12 | -190 | 219 | 144 | 1.64E-02 | 3.13E-02 |

**Table S2**: *CO adsorption and CO dissociation energies on Co(11$\bar{2}$1) $B_5$ site with and without Van der Waals correction.*

| Co(11$\bar{2}$1) $B_5$ site | | | | | |
|---|---|---|---|---|---|
| Van der Waals correction | CO adsorption energy [kJ/mol] | CO dissociation energy forward [kJ/mol] | CO dissociation energy backward [kJ/mol] | DOS average absolute deviation per data point | COHP average absolute deviation per data point |
| IVDW0 | -158 | 83 | 102 | 0.00E+00 | 0.00E+00 |
| IVDW11 | -170 | 82 | 103 | 3.70E-02 | 1.05E-01 |
| IVDW12 | -181 | 82 | 103 | 3.94E-02 | 9.39E-02 |

**Table S3**: *CO adsorption and CO dissociation energies on $Co_{52}/Al_2O_3$ NC site with and without Van der Waals correction.*

| $Co_{52}/Al_2O_3$ NC site | | | | | |
|---|---|---|---|---|---|
| Van der Waals correction | CO adsorption energy [kJ/mol] | CO dissociation energy forward [kJ/mol] | CO dissociation energy backward [kJ/mol] | DOS average absolute deviation per data point | COHP average absolute deviation per data point |
| IVDW0 | -156 | 79 | 83 | 0.00E+00 | 0.00E+00 |
| IVDW11 | -154 | 80 | 99 | 2.63E-02 | 3.27E-02 |
| IVDW12 | -165 | 81 | 100 | 3.95E-02 | 5.07E-02 |

**Table S4**: *CO adsorption and CO dissociation energies on $Co_{81}/TiO_2$ NR site with and without Van der Waals correction.*

| $Co_{81}/TiO_2$ NR site | | | | | |
|---|---|---|---|---|---|
| Van der Waals correction | CO adsorption energy [kJ/mol] | CO dissociation energy forward [kJ/mol] | CO dissociation energy backward [kJ/mol] | DOS average absolute deviation per data point | COHP average absolute deviation per data point |
| IVDW0 | -152 | 103 | 115 | 0.00E+00 | 0.00E+00 |
| IVDW11 | -166 | 104 | 109 | 2.25E-02 | 4.68E-02 |
| IVDW12 | -181 | 102 | 108 | 2.89E-02 | 5.90E-02 |

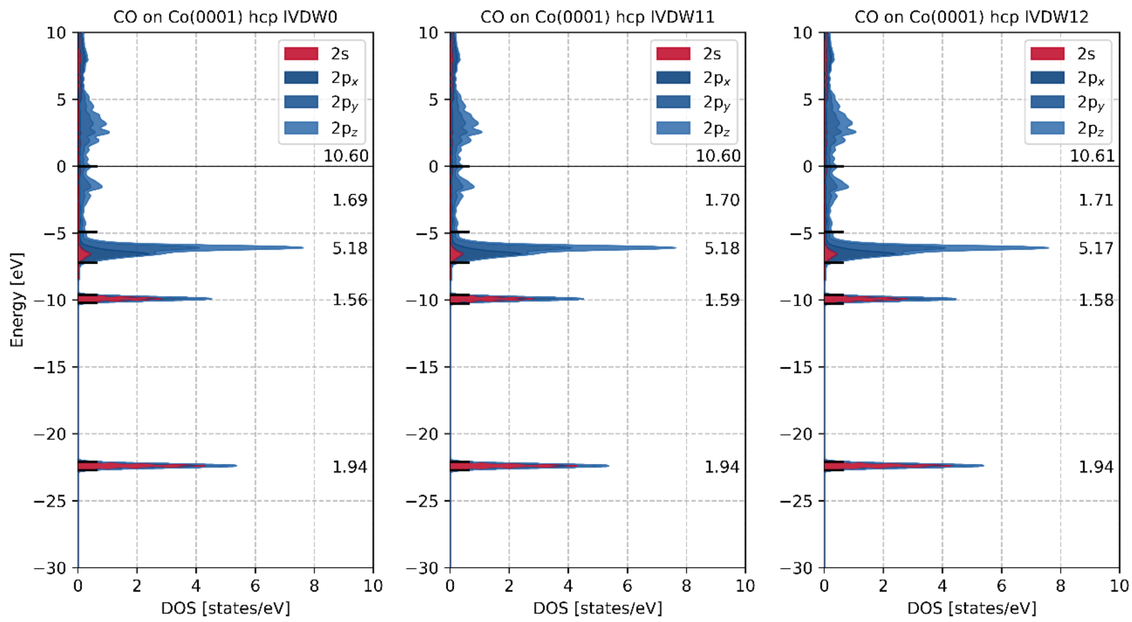


**Figure S1**: *DOS plots of predissociation state of CO on Co(0001) HCP site with and without Van der Waals correction.*

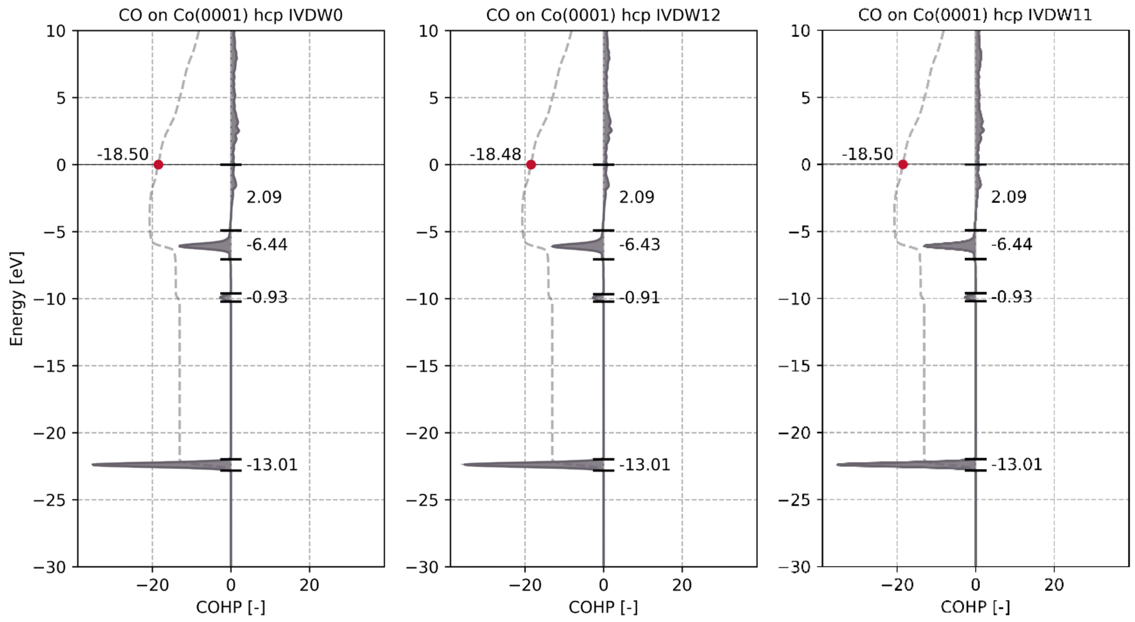


**Figure S2**: *COHP plots of predissociation state of CO on Co(0001) HCP site with and without Van der Waals correction.*

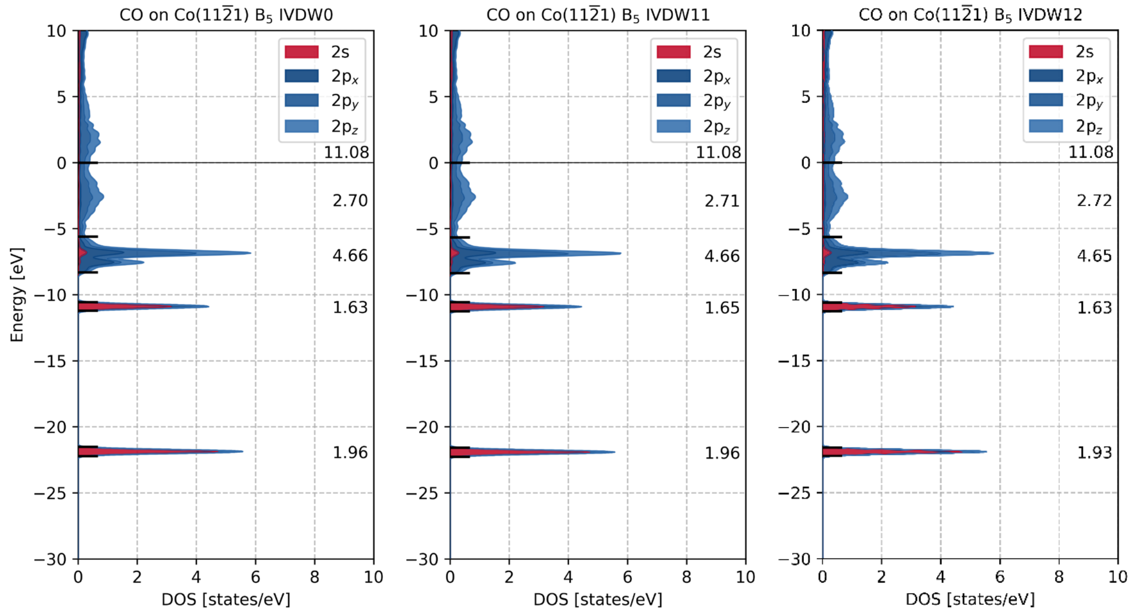


**Figure S3**: *DOS plots of predissociation state of CO on Co(11$\bar{2}$1) $B_5$ site with and without Van der Waals correction.*

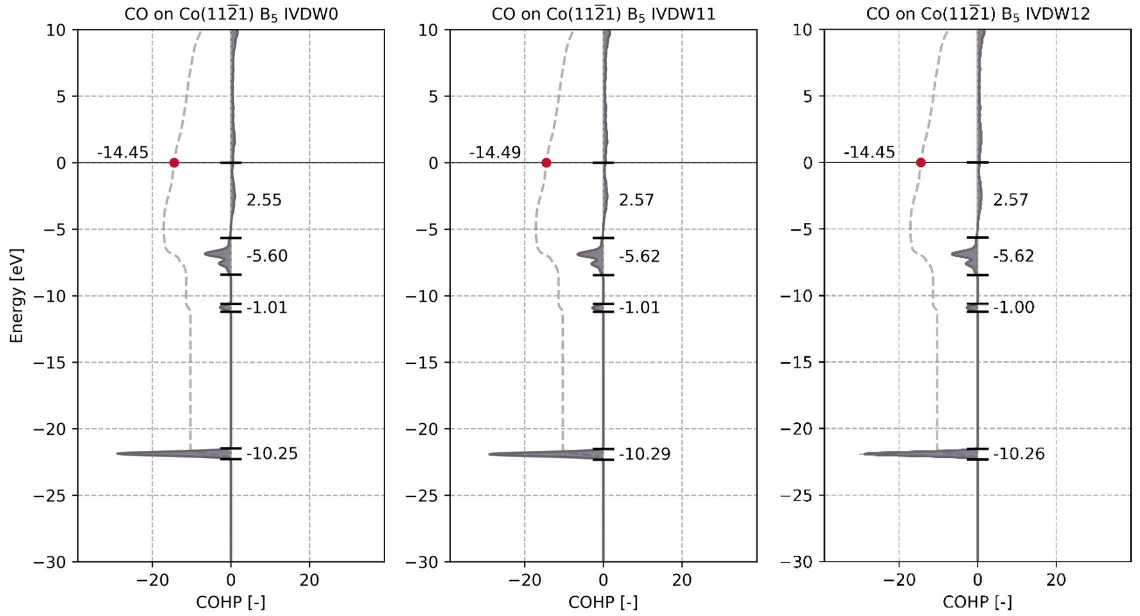


**Figure S4**: *COHP plots of predissociation state of CO on Co(11$\bar{2}$1) $B_5$ site with and without Van der Waals correction.*

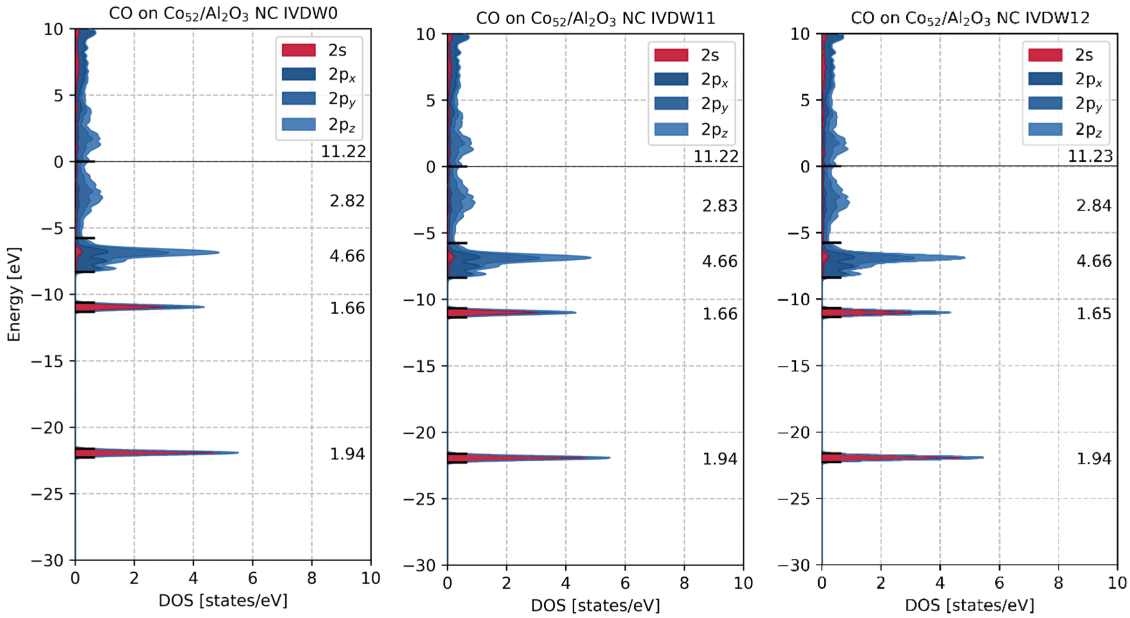


**Figure S5**: *DOS plots of predissociation state of CO on $Co_{52}/Al_2O_3$ NC site with and without Van der Waals correction.*

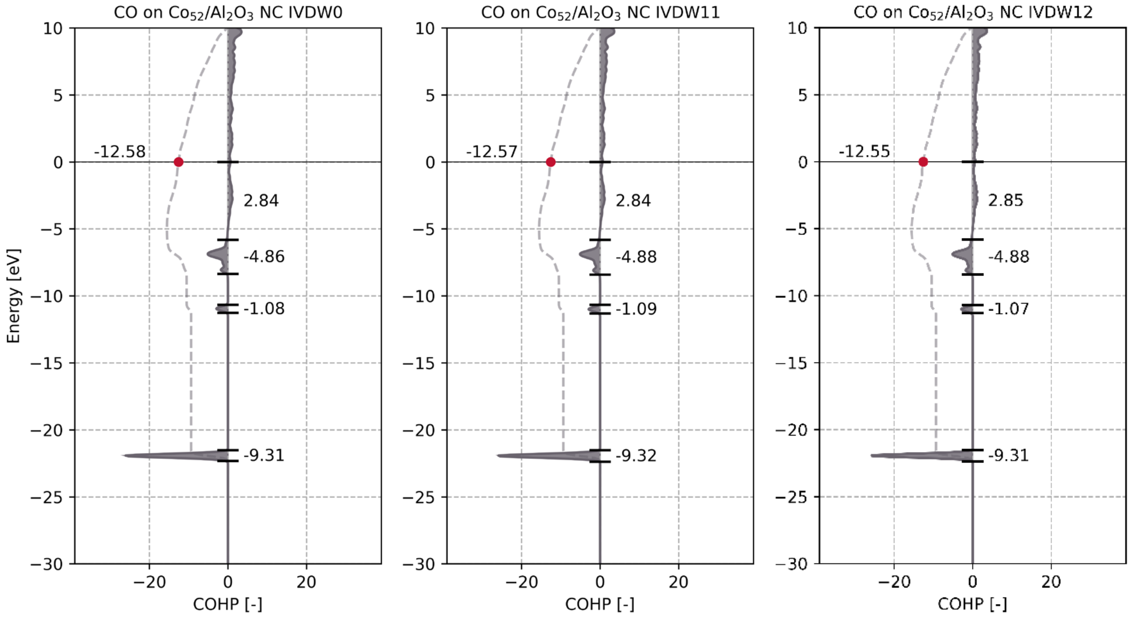


**Figure S6**: *COHP plots of predissociation state of CO on $Co_{52}/Al_2O_3$ NC site with and without Van der Waals correction.*

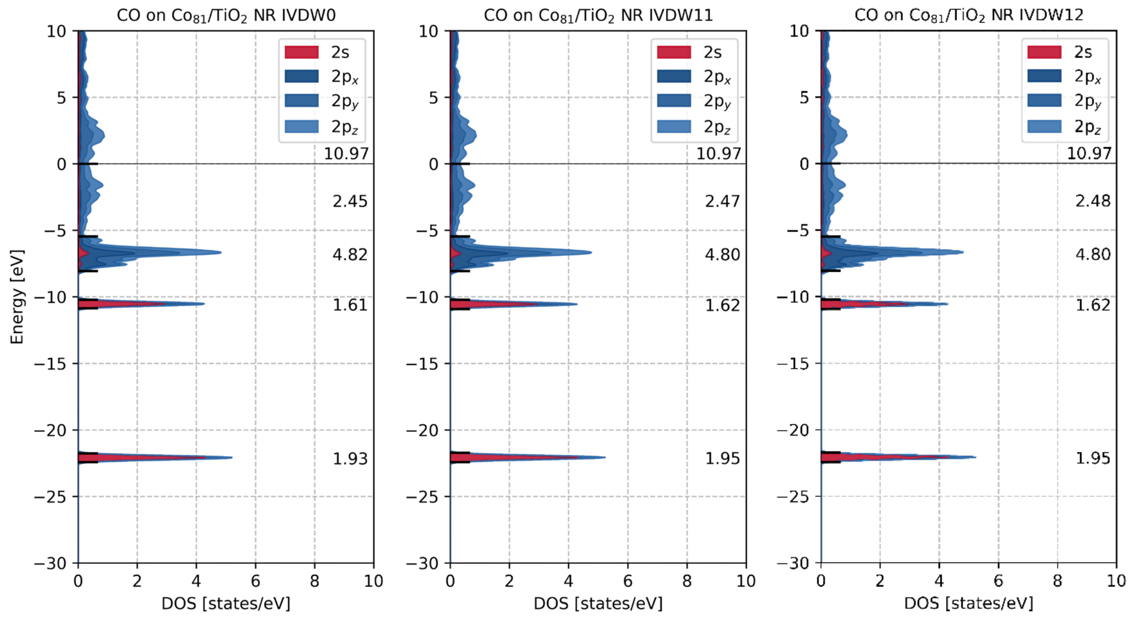


**Figure S7**: *DOS plots of predissociation state of CO on $Co_{81}/TiO_2$ NR site with and without Van der Waals correction.*

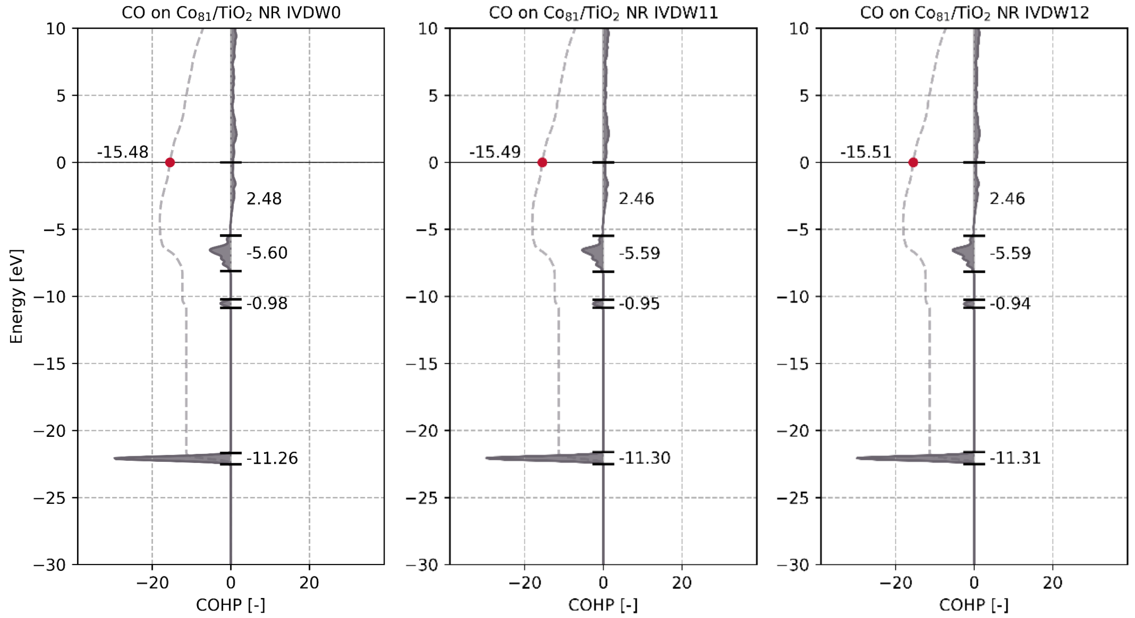


**Figure S8**: *COHP plots of predissociation state of CO on $Co_{81}/TiO_2$ NR site with and without Van der Waals correction.*

The CO adsorption energy is significantly affected by a Van der Waals correction. Generally, we see that CO adsorption becomes more stable when a Van der Waals correction is applied. The only case where this is not true is for the $Co_{52}/Al_2O_3$ NC site for the Van der Waals correction with zero-damping (IVDW11), where CO adsorbs 2 kJ/mol less stable than without correction. The largest difference between uncorrected and Van der Waals corrected values for CO adsorption is the $Co_{81}/TiO_2$ NR site for the Van der Waals correction with Becke-Johnson damping (IVDW12), with 29 kJ/mol.

For CO dissociation, the difference between uncorrected and Van der Waals corrected energies are not significant. In general, the difference is 1-2 kJ/mol. The largest difference is found for the Co(0001) HCP site, with a difference of 7 kJ/mol for IVDW12.

The DOS and COHP plots are very similar. The total DOS and COHP values deviate very little from each other, whether a Van der Waals correction was applied or not. The individual peaks are very similar as well and differ from each other by a maximum of 0.03 for the DOS peaks and 0.05 for the COHP peaks. Also, the average absolute deviation per data point is very small for each geometry. The largest value for DOS is $3.95 \times 10^{-2}$ (for $Co_{52}/Al_2O_3$ NC site) and the largest value for COHP is $1.05 \times 10^{-1}$ (for Co($11\bar{2}1$) $B_5$ site) for this metric.

From this detailed comparison we conclude that the electronic structure analyses that we have performed regarding the electronic density of CO on several Co sites and the corresponding CO dissociation energy barrier are not significantly affected by whether a Van der Waals correction is applied or not.

**Section S2**: *Type of smearing and smearing width.*

To investigate the effect of the type of smearing and the smearing width on the $Al_2O_3$ and $TiO_2$ supports, we simulated the bare $Al_2O_3$ and $TiO_2$ supports, the Co nanocluster on $Al_2O_3$, and the Co nanorod on $TiO_2$ with both Gaussian smearing, and first order method of Methfessel-Paxton smearing. For Gaussian smearing, a smearing width of smearing width of 0.05 eV was used. For the first order method of Methfessel-Paxton smearing, a smearing width of 0.2 eV was used. Since $Al_2O_3$ and $TiO_2$ are both insulators, Gaussian smearing would be the recommended choice to simulate pure $Al_2O_3$ and $TiO_2$. The differences in energy between the simulations with Gaussian smearing, and first order method of Methfessel-Paxton smearing are shown in Table S5.

**Table S5**: *Absolute energy difference between different smearing settings.*

| Model | Absolute energy difference between different smearing settings [eV] |
|---|---|
| γ-$Al_2O_3$(110) | 0.015 |
| $Co_{52}$/$Al_2O_3$ NC | 0.010 |
| rutile-$TiO_2$(110) | 0.017 |
| $Co_{81}$/$TiO_2$ NR | 0.001 |

These differences are extremely small. Therefore, we conclude that the influence of the smearing widths on the results for the $Al_2O_3$ and $TiO_2$ supports is negligible.

**Section S3**: *Performance of PBE on the cohesive energy of FCC and HCP Co.*

PBE and other GGAs are reported to perform poorly on predicting the cohesive energy of the FCC and HCP Co bulk phases.[3] We computed a cohesive energy of 5.17 and 5.19 eV/atom for Co bulk FCC and HCP, respectively. In experiments, a cohesive energy of 4.39 eV/atom is reported.[4] This difference might affect adsorption energies, however it is well-known that such adsorption energies are poorly predicted by PBE to begin with.[5] Despite the poor prediction of Co bulk energies, we assume that such errors cancel in the calculation of dissociation barriers, as indicated when using different exchange-correlation functionals or inclusion of a VdW correction. Regarding the performance of PBE on γ-$Al_2O_3$ and rutile-$TiO_2$, previous studies report that both rutile-$TiO_2$[6] and γ-$Al_2O_3$[7] are well described by the PBE functional.

**Section S4**: *Assessment of stability of supported Co nanoclusters and nanorods.*

For the $Co_{55}/Al_2O_3(110)$ nanocluster model, we took the $Ni_{55}/Al_2O_3(110)$ model from Silaghi et al.[8] The authors of this work calculated the binding energy per atom and the metal-support interaction (both defined on page S2 of the Supporting information of the work from Silaghi et al.[8]) for several $Ni_{55}$ clusters on a γ-$Al_2O_3(110)$ support, and they found the $Ni_{55}$ Marks nanocluster to be the most stable cluster of 55 Ni atoms on a γ-$Al_2O_3(110)$ support. They employed velocity scaled ab initio molecular dynamics to investigate the behavior of this nanoparticle at 1000 K and this confirmed their DFT findings. Co and Ni are next to each other in the periodic table and often show similar behavior. Therefore, we expect a $Co_{55}$ Marks nanocluster on a γ-$Al_2O_3(110)$ support to be stable as well. To confirm this, we performed calculations of one Co atom leaving the nanocluster and migrating over the $Al_2O_3$ support (detachment). For completeness, we performed these calculations for the nanocluster on the $TiO_2$ support as well, as for both nanorods on $Al_2O_3$ and $TiO_2$. The results are shown in Table S6.

**Table S6**: *Co detachment energy of supported Co nanoclusters and nanorods.*

| Model | Co detachment energy [kJ/mol] |
|---|---|
| $Co_{55}/Al_2O_3$ nanocluster | 317 |
| $Co_{84}/Al_2O_3$ nanorod | 554 |
| $Co_{54}/TiO_2$ nanocluster | 90 |
| $Co_{81}/TiO_2$ nanorod | 106 |

The energies in Table S6 show that an event where a Co atom detaches from the nanoclusters or nanorods is endothermic. The Co detachment energies are quite high, indicating that this is not likely to happen. We observe that a Co atom is more stabilized on the $TiO_2$ support than on the $Al_2O_3$ support in line with known strong metal-support interactions[9], giving rise to lower Co detachment energies for the nanocluster and nanorod on the $TiO_2$ support. For both supports, the Co detachment energy for the nanocluster is lower than for the nanorod.

**Section S5**: *Dipole correction.*

For the Co slabs, we added a large vacuum layer of at least 15 Å to avoid dipole interactions between the adsorbates. For the supported Co nanorods and nanoclusters, where the vacuum layers are smaller than 15 Å (but larger than 12 Å), we tested whether applying a dipole correction resulted in different adsorption and dissociation energies. For the Co/$Al_2O_3$ nanocluster and the Co/$TiO_2$ nanorod, we computed the differences in CO adsorption energy, forward and backward CO dissociation energy. The results are shown in Table S7. The differences are found to be sufficiently small to discard the use of dipole corrections for these systems.

**Table S7**: *Absolute energy difference between calculations with and without dipole correction.*

| Model | Difference in CO adsorption energy [eV] | Difference in CO dissociation energy forward [eV] | Difference in CO dissociation energy backward [eV] |
|---|---|---|---|
| $Co_{52}/Al_2O_3$ pocket site | 0.00 | 0.01 | 0.00 |
| $Co_{NR}/TiO_2$ pocket site | 0.02 | 0.04 | 0.05 |

**Section S6**: *Comparison between PBE and RPBE.*

As mentioned in the Methods section, we use the PBE functional for the exchange-correlation in our DFT calculations. Literature shows that CO adsorption is highly dependent on choice of exchange-correlation functional.[10] To assess the dependence of our results on the choice of exchange-correlation functional, we calculated a few systems with the revised PBE from Hammer et al.[11] The results are summarized in Table S8.

**Table S8**: *Comparison of CO adsorption energies, forward and backward energies for CO dissociation between PBE and revised PBE from Hammer et al.*
*The states and barriers are not corrected for zero-point energy.*

| | CO adsorption energy [kJ/mol] | | | CO dissociation barrier forward [kJ/mol] | | | CO dissociation energy backward [kJ/mol] | | |
|---|---|---|---|---|---|---|---|---|---|
| Model | PBE | RPBE | Δ | PBE | RPBE | Δ | PBE | RPBE | Δ |
| Co($11\bar{2}1$) $B_5$ | -158 | -22 | 136 | 83 | 88 | 4 | 102 | 97 | -5 |
| $Co_{52}/Al_2O_3$ NC | -155 | -17 | 139 | 79 | 80 | 1 | 83 | 90 | 7 |
| $Co_{54}/TiO_2$ NC | -167 | -24 | 142 | 91 | 89 | -2 | 144 | 160 | 16 |

The difference in adsorption energies between PBE and RPBE are very large: approximately 140 kJ/mol. It known that PBE overestimates the CO adsorption energy.[10] For the barriers, the difference between PBE and RPBE is much smaller. The absolute differences between the barriers computed with PBE and RPBE lie between 1 and 4 kJ/mol. This is well within accuracy limit of DFT (10 kJ/mol). The backward dissociation barrier for $Co_{54}/TiO_2$ NC shows a difference of 16 kJ/mol between PBE and RPBE. The forward CO dissociation barriers thus are likely to be independent of the used exchange-correlation functional.

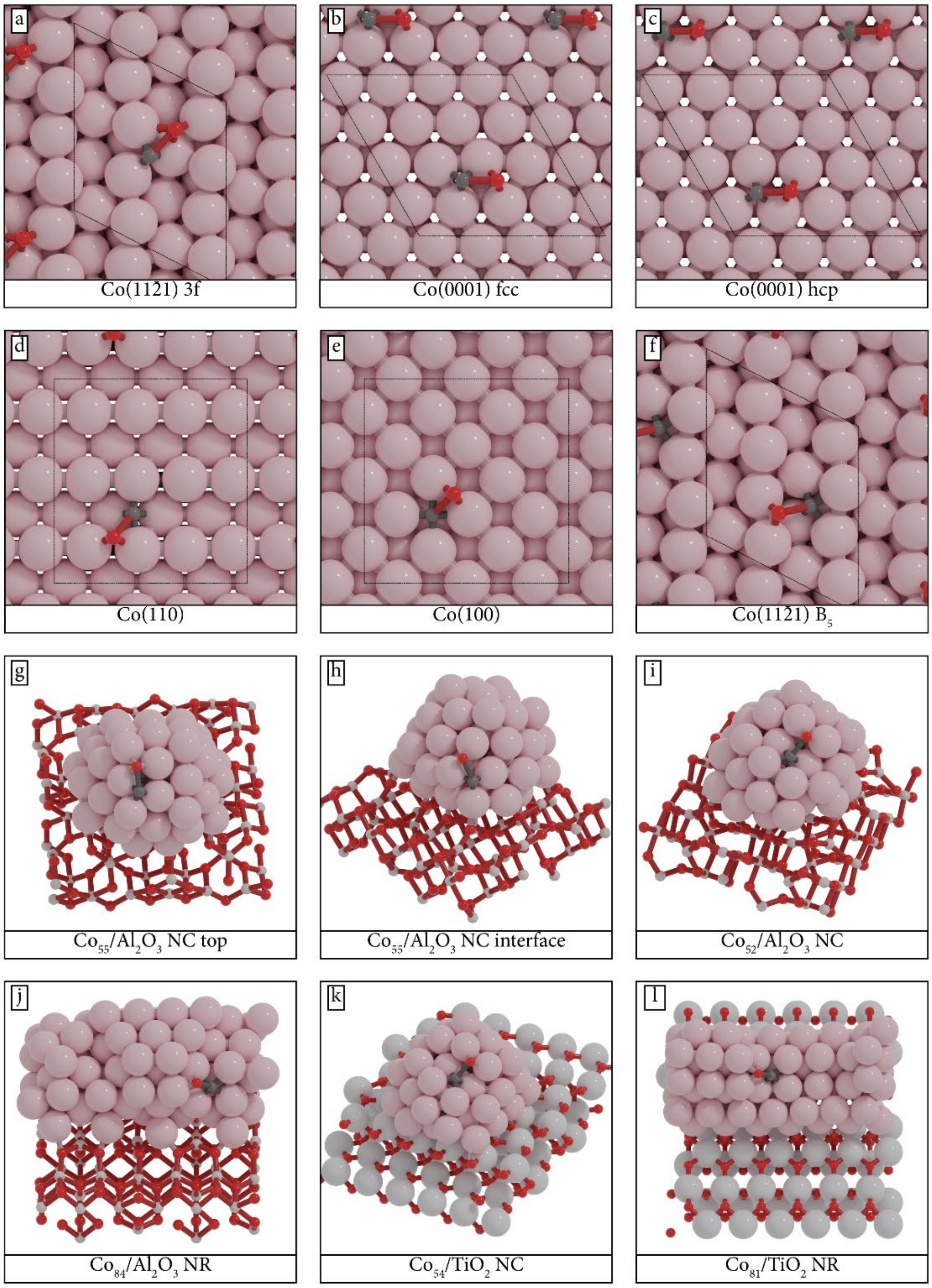


**Figure S9**: *Geometry of transition state of CO dissociation on twelve Co sites.*

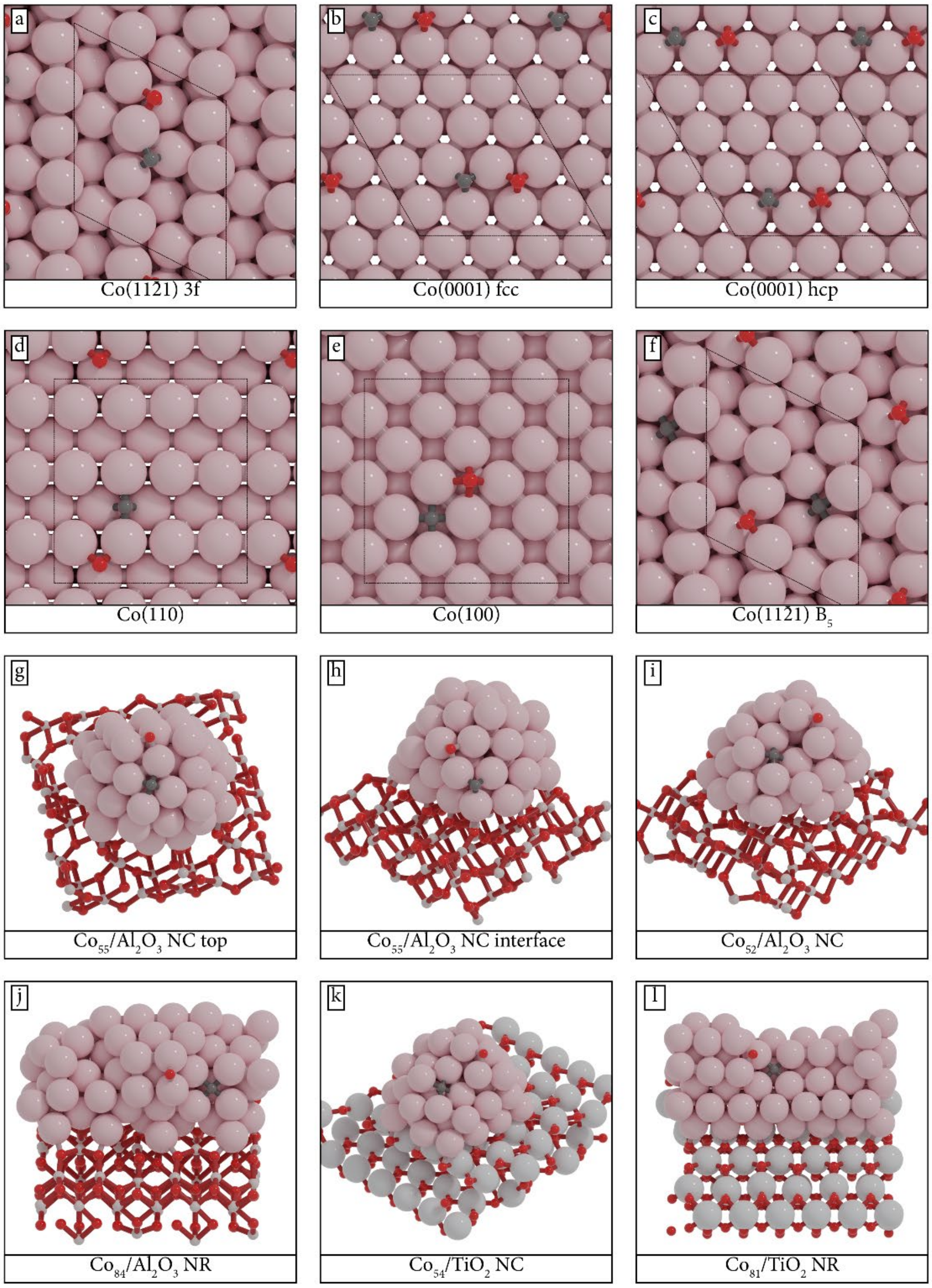


**Figure S10**: *Geometry of final state of CO dissociation on twelve Co sites.*

**Section S7:** *Description of initial, transition and final states of CO dissociation.*
First, we compare the six extended surfaces (Figure 1a-f). In Figure 1a, CO adsorbs on a threefold site as exposed on the Co($11\bar{2}1$) surface. The adsorption energy is -166 kJ/mol. In the transition state, the oxygen moiety migrates to a neighboring active site and is bonded to the surface in a quasi-threefold configuration. The carbon atom remains in the threefold site. In the transition state, C and O share two Co atoms. In the final state, the oxygen atom continues to migrate away from the carbon atom and adsorbs at an adjacent threefold site. Only a single Co atom is shared between C and O. This elementary reaction step has an activation energy of 242 kJ/mol and is endothermic by 112 kJ/mol.

In Figure 1b, the initial state of CO adsorbed on an FCC site on the Co(0001) is shown. This state has an adsorption energy of -158 kJ/mol. CO dissociation proceeds by the CO moiety bending towards the surface by which O adsorbs in a bridge configuration. In this configuration, the C and O atoms share a single Co atom. In the final state, the oxygen atom has migrated to an adjacent FCC site and resides in a threefold configuration. Like the transition state, also in the final state only a single Co atom is shared between C and O. The dissociation reaction has a barrier of 229 kJ/mol and is endothermic by 119 kJ/mol.

Figure 1c shows CO adsorbed on an HCP site on the Co(0001) surface, in a threefold fashion. The adsorption is exothermic by 160 kJ/mol. As on the Co(0001) FCC site, in transition state the oxygen adsorbs in a bridged manner on the surface, while the carbon remains in its place. One cobalt atom is shared with carbon in this state. In the final state, the C and O occupy two neighboring HCP threefold sites, while sharing one cobalt atom. This dissociation step has an energy barrier of 222 kJ/mol and is endothermic by 87 kJ/mol.

In Figure 1d, CO adsorbs on the Co(110) surface with an adsorption energy of -135 kJ/mol. In the adsorbed state, carbon is bonded to four cobalt atoms. In the transition state, the oxygen attaches in a bridge manner to two cobalt atoms. One cobalt atom is shared between oxygen and carbon. Carbon remains at its fourfold position. The oxygen moves further between the two cobalt atoms until it can bond to a third cobalt atom in the final state. One cobalt atom is shared by C and O. The energy barrier associated with this reaction step is 116 kJ/mol. This step is endothermic by 11 kJ/mol.

Figure 1e displays CO adsorbed at a fourfold site on the Co(100) surface, which corresponds to an energy of -175 kJ/mol. To dissociate, oxygen moves to a bridged position between two cobalt atoms. Both these cobalt atoms are also bonded to carbon. Carbon stays in its original place in the transition state. In the final state, O is adsorbed at a fourfold site next to the adsorption site of C. The energy barrier of this step is 125 kJ/mol, and the reaction step is exothermic by 43 kJ/mol.

Figure 1f displays the Co($11\bar{2}1$) surface, which possesses a $B_5$ site that accommodates a sixfold adsorption of CO, with carbon and oxygen atoms bonding to the metal in a fourfold and twofold configuration, respectively. Notably, carbon and oxygen atoms do not share any cobalt atoms in this initial state, and the adsorption energy is -163 kJ/mol. The transition state exhibits an elongated C-O bond due to the movement of O away from C while C remains stationary, with an activation energy of 82 kJ/mol. Subsequently, oxygen moves further between two cobalt atoms to bond with a third cobalt atom in the final state, where C and O atoms continue to not share any cobalt atoms. This CO dissociation process is slightly exothermic, with an energy release of 19 kJ/mol.

Next, we compare the six supported nanoclusters and -rods as shown in Figure 1g-l. Figure 1g depicts a threefold top site on a $Al_2O_3$-supported nanocluster of 55 cobalt atoms. The energy of adsorption on this site is -169 kJ/mol. Because of the low coordination numbers of the Co atoms, they are relatively mobile. Upon CO dissociation, this enhanced mobility allows for the emergence of an adjacent

threefold site accepting the oxygen moiety in the transition state. In this configuration, one Co is shared between C and O. As oxygen moves further away from carbon, the cobalt atoms perturb again in such a way that a fourfold site harboring the carbon is formed. Oxygen is positioned at the adjacent threefold site in the final state. One cobalt atom is bonded to both C and O. This elementary reaction step has an activation energy of 201 kJ/mol and is endothermic by 36 kJ/mol.

In Figure 1h, CO is adsorbed on a threefold site close to the support of the $Co_{55}/Al_2O_3$ model. One of the three cobalt atoms of the threefold site is attached directly to the support. CO adsorbs on this site with -180 kJ/mol. Oxygen moves away from carbon until it is positioned in a bridged fashion between two cobalt atoms. One of these cobalt atoms is also bonded to carbon. In the final state, the oxygen also bonds to a third Co atom that is not bonded to carbon. The Co atoms do not perturb as extensively as observed for the $Co_{55}/Al_2O_3$ system. This reaction has a barrier of 206 kJ/mol and is endothermic by 50 kJ/mol.

Figure 1i shows the adsorption of CO on a pocket site of the $Co_{52}/Al_2O_3$ model. Both carbon (fourfold) and oxygen (twofold) bond to the metal in the initial state very much akin to the fourfold adsorption mode found for the Co($11\bar{2}1$) model (Figure 1f). The adsorption energy is -152 kJ/mol. No cobalt atoms are shared between C and O. The transition state resembles the initial state, but with an increased C-O bond distance. In the final state, the C-O distance increases further, and O adsorbs in a threefold mode to a site neighboring the fourfold site on which carbon is still adsorbed. No cobalt atoms are shared between oxygen and carbon in this final state. The barrier for this dissociation is 75 kJ/mol and the dissociation is slightly exothermic by 4 kJ/mol. Notably, this barrier is lower in comparison to the situation found for the similar situation on the extended Co($11\bar{2}1$) model.

The $Al_2O_3$-supported nanorod in Figure 1j exhibits a site close to the support on which CO can adsorb in a fivefold manner. Carbon bonds in a fourfold manner, oxygen bonds to a single cobalt atom that is not shared with carbon. The adsorption energy is -156 kJ/mol. Oxygen moves towards a bridged position between the cobalt atom to which it was attached in initial state and a cobalt atom that carbon bonds to. In this transition state, carbon remains fourfold bonded to cobalt. In the final state, oxygen bonds in a bridged manner between two cobalt atoms that are not shared with carbon. The barrier for this dissociation is 121 kJ/mol. The dissociation is exothermic by 17 kJ/mol.

In Figure 1k, CO adsorbs in a sixfold manner in a pocket site on a $TiO_2$-supported nanocluster. The adsorption energy is -158 kJ/mol. The initial, transition and final states are similar to the initial, transition and final states on the $Al_2O_3$-supported pocket site in Figure 1i. A noticeable difference is the higher mobility of the cobalt atoms in Figure 1k. In the final state, the cobalt atoms have a different configuration than in the initial state, with carbon's fourfold and oxygen's threefold neighboring sites shifted with respect to each other. The barrier that belongs to this elementary reaction step is 78 kJ/mol. The reaction is exothermic by 18 kJ/mol.

Figure 1l shows CO on a $TiO_2$-supported nanorod. The initial state is like Figure 1j, with an adsorption energy of -152 kJ/mol. The transition state is also like Figure 1j, but oxygen bonds to an extra Co atom compared to Figure 1j. This Co atom does not bond to C. The final state resembles Figure 1j as well, but instead of only bonding bridged between two Co atoms that are not shared with C, O bonds also to a third Co atom which is shared with C. The activation energy for this CO dissociation is 100 kJ/mol and the reaction energy is -10 kJ/mol.

**Table S9**: *Forward and backward CO dissociation energies for the extended surface models and the values reported in the literature.*

| Model | CO dissociation barrier forward [kJ/mol] | CO dissociation energy backward [kJ/mol] | Source |
|---|---|---|---|
| Co($11\bar{2}1$) 3f | 242 | 130 | This work |
| Co(0001) FCC | 229 | 110 | This work |
| Co(0001) HCP | 222 | 135 | This work |
| Co(110) | 116 | 105 | This work |
| Co(100) | 125 | 168 | This work |
| Co($11\bar{2}1$) $B_5$ | 82 | 101 | This work |
| Co(0001) | 225 | 158 | [12] |
| Co(0001) | 233 | - | [13] |
| Co(0001) | 235 | 190 | [14] |
| Co(110) | 135 | - | [15] |
| Co(100) | 133 | - | [15] |
| Co(100) | 131 | 199 | [14] |
| Co($11\bar{2}1$) $B_5$ | 100 | 102 | [12] |

**Section S8:** *Approximating orbital overlap between CO and Co.*

Using the atom positions from the with DFT-optimized predissociation states, we calculated the electron density overlap between CO and Co as if their electron densities were only radially dependent. We computed the overlap $\mu_2$ as defined in Equation 2 with $\phi(r) = \exp(-\alpha \cdot r)$ (Figure S11c) and $\phi(r) = \exp(-\alpha \cdot r^2)$ (Figure S11d). This was done by placing a Lebedev quadrature grid on the carbon and oxygen atoms. By looping over all cobalt atoms, the overlap for each cobalt atom and the carbon or oxygen atom was determined according to the formula for $\phi$. The sum of the overlap equals the overlap metric $\mu_2$. To find the best correlation, the fitting parameter $\alpha$ was optimized for both $\phi(r)$ functions. This was done by minimizing the Pearson correlation coefficient with the Nelder-Mead method.

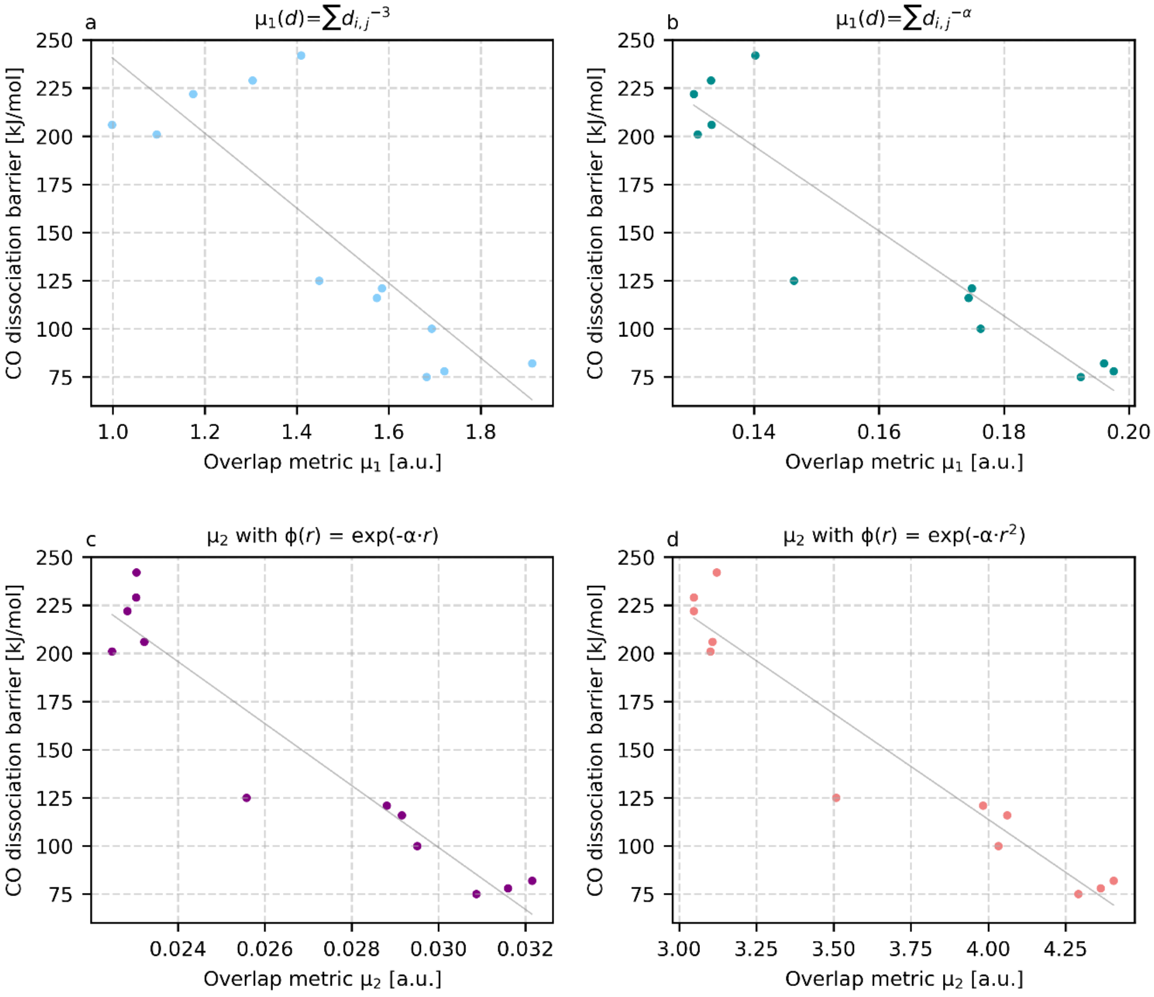


**Figure S11**: *Correlation between CO dissociation barrier and the electron density overlap between CO and Co in the predissociation state.*

*Distance and overlap metrics are computed with* $\mu_1(d) = \sum_{i \in Co, j \in C,O} {d_{i,j}}^{-3}$*, with a Pearson correlation coefficient of -0.91 (a);* $\mu_1(d) = \sum_{i \in Co, j \in C,O} {d_{i,j}}^{-\alpha}$ *with* $\alpha = 4.9$ *for C-Co distances and* $\alpha = 6.4$ *for O-Co distances, with a Pearson correlation coefficient of -0.93 (b);* $\mu_2 = \sum_{i \in Co, j \in C,O} \langle \phi_i | \phi_j \rangle$ *with* $\phi(r) = \exp(-\alpha \cdot r)$ *with* $\alpha = 2.0$ *for C and O, and* $\alpha = 5.4$ *for Co, with a Pearson correlation coefficient of -0.95 (c);* $\mu_2 = \sum_{i \in Co, j \in C,O} \langle \phi_i | \phi_j \rangle$ *with* $\phi(r) = \exp(-\alpha \cdot r^2)$ *with* $\alpha = 0.97$ *for C, O, and* $\alpha = 0.75$ *for Co, with a Pearson correlation coefficient of -0.96 (d).*

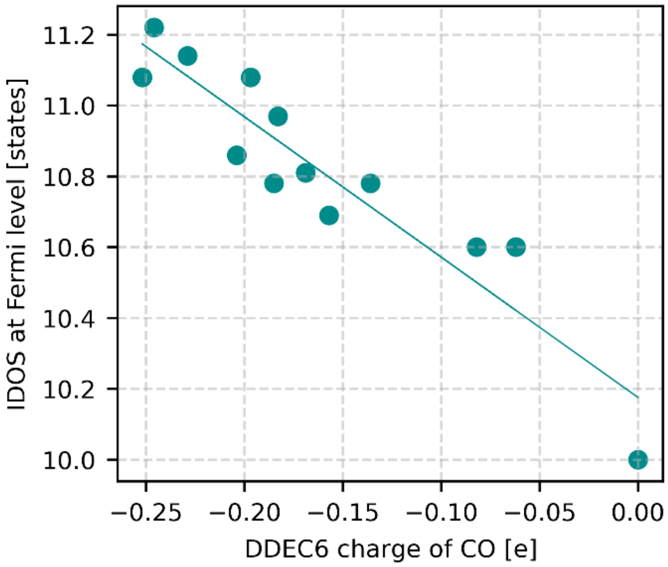


**Figure S12**: *Correlation between IDOS at Fermi level and the DDEC6 charge of adsorbed CO. The Pearson correlation coefficient for this correlation is -0.93.*

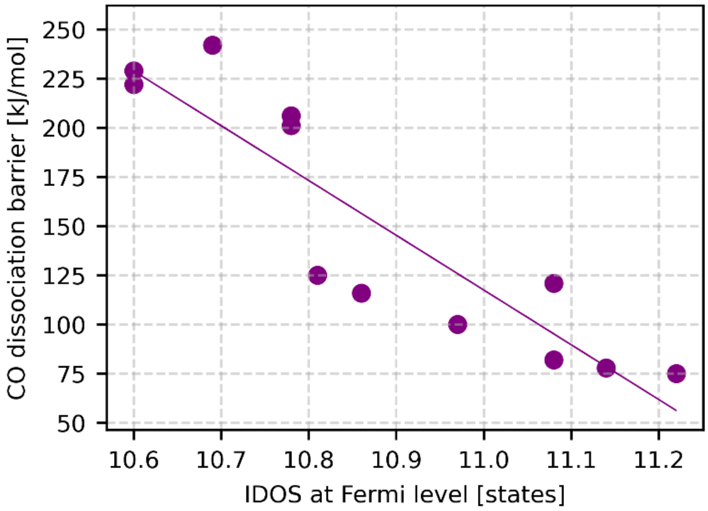


**Figure S13**: *Correlation between CO dissociation barrier and IDOS at Fermi level. The Pearson correlation coefficient for this correlation is -0.91.*

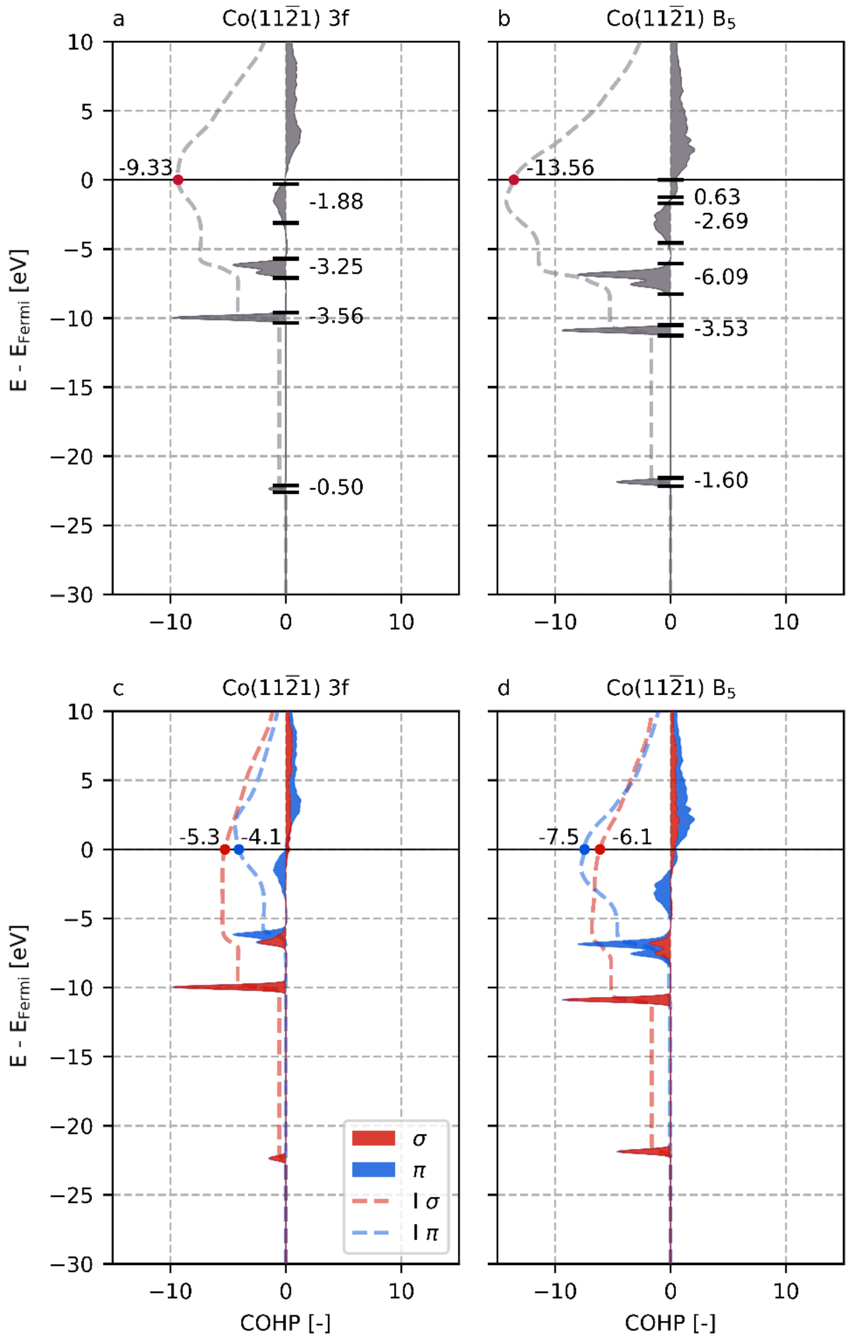


**Figure S14**: *COHP as function of the energy of the Kohn-Sham states of the Co-CO bond for CO adsorbed on Co(11$\bar{2}$1) 3f (a,c), and CO adsorbed on Co(11$\bar{2}$1) B$_5$ (b,d).*
*Subfigures a and b show the total and integrated COHP for CO, whereas subfigures c and d show the σ- and π-contributions. All plots use the same reference energy. The integrated COHP at Fermi level is shown above the black line at zero energy (a,b), or near the red and blue dots (c,d). The values next to the peaks pertain to the area under the curves. The dashed lines show the integrated σ- and π-contributions (I σ and I π).*

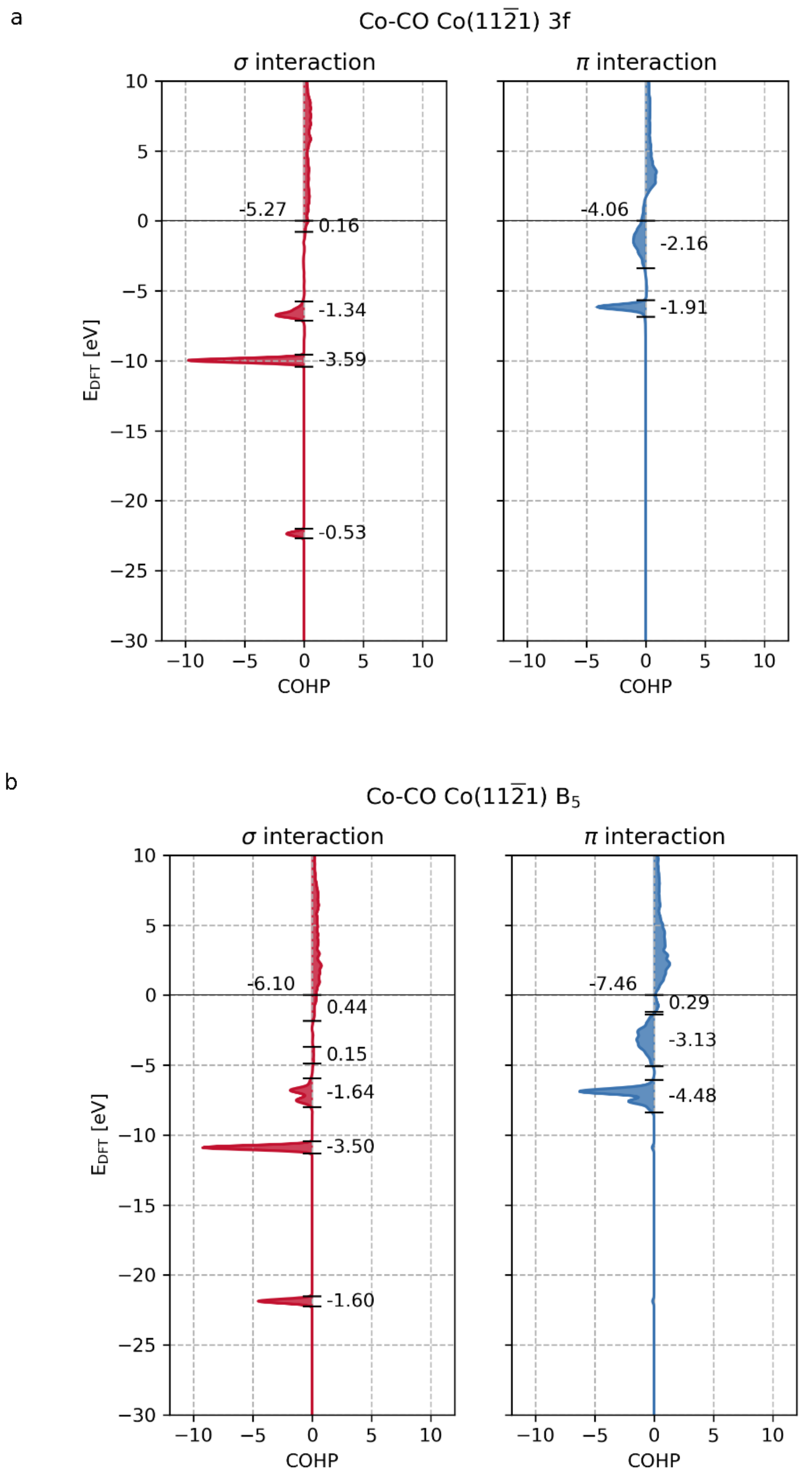


**Figure S15**: *σ- and π-contributions of the COHP as function of the energy of the Kohn-Sham states of the Co-CO bond for CO adsorbed on Co(11$\bar{\mathbf{2}}$1) 3f (a), and CO adsorbed on Co(11$\bar{\mathbf{2}}$1) $B_5$ (b).*
*All plots use the same reference energy. The integrated COHP at Fermi level is shown above the black line at zero energy.*

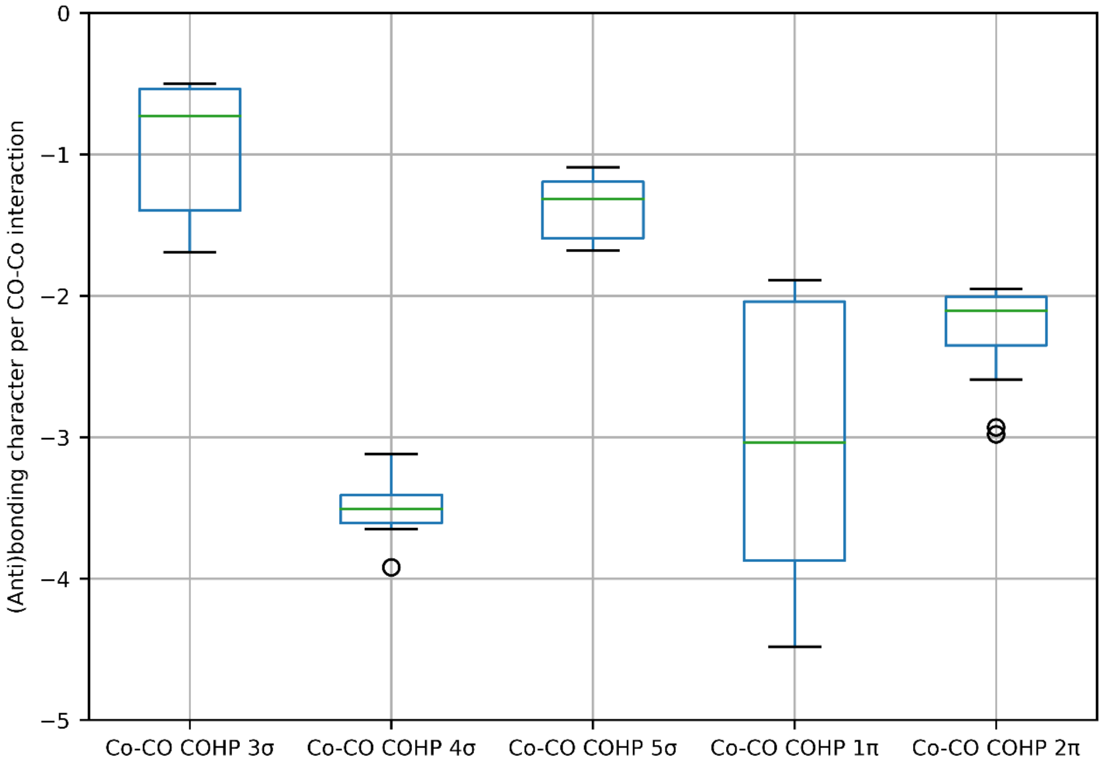


**Figure S16**: *Boxplot of the COHP values of the Co-CO interactions.*

## References


(1) Grimme, S.; Antony, J.; Ehrlich, S.; Krieg, H. A Consistent and Accurate *Ab Initio* Parametrization of Density Functional Dispersion Correction (DFT-D) for the 94 Elements H-Pu. *J. Chem. Phys.* **2010**, *132* (15), 154104. https://doi.org/10.1063/1.3382344.

(2) Grimme, S.; Ehrlich, S.; Goerigk, L. Effect of the Damping Function in Dispersion Corrected Density Functional Theory. *J. Comput. Chem.* **2011**, *32* (7), 1456–1465. https://doi.org/10.1002/jcc.21759.

(3) De La Peña O'Shea, V. A.; Moreira, I. D. P. R.; Roldán, A.; Illas, F. Electronic and Magnetic Structure of Bulk Cobalt: The α, β, and ε-Phases from Density Functional Theory Calculations. *J. Chem. Phys.* **2010**, *133* (2), 024701. https://doi.org/10.1063/1.3458691.

(4) Taylor, A. Lattice Parameters of Binary Nickel-Cobalt Alloys. *J. Inst. Met.* **1950**, *77*, 585–594.

(5) Feibelman, P. J.; Hammer, B.; Nørskov, J. K.; Wagner, F.; Scheffler, M.; Stumpf, R.; Watwe, R.; Dumesic, J. The CO/Pt(111) Puzzle. *J. Phys. Chem. B* **2001**, *105* (18), 4018–4025. https://doi.org/10.1021/jp002302t.

(6) Mohamad, M.; Ul Haq, B.; Ahmed, R.; Shaari, A.; Ali, N.; Hussain, R. A Density Functional Study of Structural, Electronic and Optical Properties of Titanium Dioxide: Characterization of Rutile, Anatase and Brookite Polymorphs. *Mater. Sci. Semicond. Process.* **2015**, *31*, 405–414. https://doi.org/10.1016/j.mssp.2014.12.027.

(7) Saelee, T.; Apichoksiri, P.; Rittiruam, M.; Wangphon, C.; Khajondetchairit, P.; Praserthdam, S.; Praserthdam, P. A Density Functional Theory Study on How γ-$Al_2O_3$ – Boehmite Transformation Affects Carbon Evolution during Aqueous-Phase Reaction. *Chemosphere* **2023**, *340*, 139842. https://doi.org/10.1016/j.chemosphere.2023.139842.

(8) Silaghi, M.-C.; Comas-Vives, A.; Copéret, C. $CO_2$ Activation on Ni/γ–$Al_2O_3$ Catalysts by First-Principles Calculations: From Ideal Surfaces to Supported Nanoparticles. *ACS Catal.* **2016**, *6* (7), 4501–4505. https://doi.org/10.1021/acscatal.6b00822.

(9) Van Etten, M. P. C.; De Laat, M. E.; Hensen, E. J. M.; Filot, I. A. W. Unraveling the Role of Metal–Support Interactions on the Structure Sensitivity of Fischer–Tropsch Synthesis. *J. Phys. Chem. C* **2023**, *127* (31), 15148–15156. https://doi.org/10.1021/acs.jpcc.3c02240.

(10) Wellendorff, J.; Silbaugh, T. L.; Garcia-Pintos, D.; Nørskov, J. K.; Bligaard, T.; Studt, F.; Campbell, C. T. A Benchmark Database for Adsorption Bond Energies to Transition Metal Surfaces and Comparison to Selected DFT Functionals. *Surf. Sci.* **2015**, *640*, 36–44. https://doi.org/10.1016/j.susc.2015.03.023.

(11) Hammer, B.; Hansen, L. B.; Nørskov, J. K. Improved Adsorption Energetics within Density-Functional Theory Using Revised Perdew-Burke-Ernzerhof Functionals. *Phys. Rev. B* **1999**, *59* (11), 7413–7421. https://doi.org/10.1103/PhysRevB.59.7413.

(12) Zijlstra, B.; Broos, R. J. P.; Chen, W.; Filot, I. A. W.; Hensen, E. J. M. First-Principles Based Microkinetic Modeling of Transient Kinetics of CO Hydrogenation on Cobalt Catalysts. *Catal. Today* **2020**, *342*, 131–141. https://doi.org/10.1016/j.cattod.2019.03.002.

(13) Ge, Q.; Neurock, M. Adsorption and Activation of CO over Flat and Stepped Co Surfaces: A First Principles Analysis. *J. Phys. Chem. B* **2006**, *110* (31), 15368–15380. https://doi.org/10.1021/jp060477i.

(14) Chen, C.; Wang, Q.; Zhang, R.; Hou, B.; Li, D.; Jia, L.; Wang, B. High Coverage CO Adsorption and Dissociation on the Co(0001) and Co(100) Surfaces from DFT and Thermodynamics. *Appl. Catal. A: General* **2016**, *523*, 209–220. https://doi.org/10.1016/j.apcata.2016.06.013.

(15) Van Etten, M. P. C.; Zijlstra, B.; Hensen, E. J. M.; Filot, I. A. W. Enumerating Active Sites on Metal Nanoparticles: Understanding the Size Dependence of Cobalt Particles for CO Dissociation. *ACS Catal.* **2021**, *11* (14), 8484–8492. https://doi.org/10.1021/acscatal.1c00651.
